# What Can We Learn Four Years On? A Multi‐Centre Service Evaluation Exploring Symptoms, Functional Impact, Recovery and Care Pathways in Long Covid

**DOI:** 10.1111/hex.70435

**Published:** 2025-11-06

**Authors:** Cassie Lee, Paul Williams, Amiad Abrahams, Julie Darbyshire, Helen E. Davies, Johannes De Kock, Umut Esmer, Samantha A. Jones, Vicky Newey, Janet Scott, Nikki Smith, Darren Winch, Harsha Master, Sarah Elkin, LOCOMOTION Consortium

**Affiliations:** ^1^ Imperial College Healthcare NHS Trust London UK; ^2^ Hertfordshire Community NHS Trust Hertfordshire UK; ^3^ Central and Northwest London NHS London UK; ^4^ Nuffield Department of Primary Care Health Sciences The University of Oxford Oxford UK; ^5^ Department of Respiratory Medicine Cardiff and Vale University Hospital Board, University Hospital Llandough, University Hospital of Wales Cardiff UK; ^6^ NHS Highland Inverness UK; ^7^ NWU School for Psychosocial Research South Africa; ^8^ Person with Long Covid UK; ^9^ Development and Innovation Department NHS Highland Inverness UK; ^10^ MRC‐University of Glasgow Centre for Virus Research Glasgow UK; ^11^ National Heart and Lung Institute, Imperial College London UK

**Keywords:** Covid‐19, long Covid, long‐term conditions, mixed‐methods, post‐Covid syndrome, service evaluation, user experience

## Abstract

**Background:**

Long Covid (LC) is associated with long‐term health impacts that require ongoing support from healthcare services. We aimed to gain insights into patients' perceptions of their ongoing symptoms of LC, the effect on daily living and vocation, perceptions of what helps with LC recovery, as well as LC care pathways and ongoing care needs.

**Methods:**

An online survey was sent to 513 participants who had used one of three LC services across England and Wales between 2020 and 2024. Participants were invited to share their experiences. We employed a mixed‐methods approach for data analysis, synthesising findings from quantitative and qualitative data. All data shared between sites was de‐identified.

**Results:**

269 (52.4%) participants completed the survey. The mean age was 52.7 (sd ± 12.0), 69.1% female and 55.0% were White‐British. The mean duration since initial SARS‐CoV‐2 infection was over 3 years (1204.4 ± 275.7 days). The Post‐Covid Functional Status (PCFS) scale indicated that most participants (94.1%, *n* = 253) had not fully recovered. When employing a global rate of change scale, 39.0% (*n* = 96) of 246 responders indicated they are still making improvements with respect to their recovery; 40.7% (*n* = 100) had plateaued, and 20.3% (*n* = 50) reported a worsening trajectory. Those with ongoing symptoms described fatigue 83.0% (*n* = 210), cognitive dysfunction 58.5% (*n* = 148) and breathlessness or wheezing 43.9% (*n* = 111) most frequently. Of those who responded, 20.8% (*n* = 48) were ‘working as prior to their initial LC infection’ and 25.5% (*n* = 59) were currently ‘unable to work’. Almost half (44.3%; *n* = 86) were no longer receiving care whilst also reporting unmet care needs. In total, 62.7% (*n* = 126) of participants indicated unmet care needs, and qualitative analysis indicated five overarching domains as having an important impact on long‐term LC recovery and ongoing healthcare needs. These were Living with LC, LC interventions and recovery, Approach to the delivery of care, Insufficient support and Suggestions and improvement.

**Conclusion:**

This study indicates the extent to which individuals continue to experience ongoing symptoms of LC, including aspects related to recovery and vocational impact, highlighting the potential widening gap between the ongoing need to support those living with LC and the limited provision of care.

**Patient or Public Contribution:**

The survey was co‐produced with Experts by Experience, who had previously attended an LC service and members of the Patient Advisory Group within the Locomotion Consortium. Collaboration and involvement continued throughout the study, including analysis, interpretation and writing processes.

**Clinical Trial Registration:**

Study registration details are available at ClinicalTrials.gov: NCT05057260 and ISRCTN: 15022307.

## 1 Introduction

Long Covid (LC) refers to the symptoms beyond 12 weeks following SARS‐CoV‐2 infection, with no alternative explanation [1, 2]. This multi‐system condition can manifest as a wide range of physical symptoms, including fatigue, breathlessness, palpitations, joint/muscle pain, gastric symptoms, neurocognitive and psychological symptoms including attention and memory alterations, anxiety and depression [3, 4]. The clinical presentation and impact of symptoms can vary and fluctuate or relapse over time [5].

True prevalence is unknown; estimates in the United Kingdom vary between 3% and 6% of the population [6]. As of April 2024, the UK Office for National Statistics reported 3.3% (2 million) people in England and Scotland were living with LC, of which 19.2% report a negative impact on their ability to undertake daily activities [6, 7]. At a similar time, the United States reported approximately 5%–6% of adults report living with LC, with a quarter experiencing significant functional limitation [8]. These ongoing symptoms and impact present a national and global healthcare demand.

In response, to support the diagnosis and management of those living with LC, the UK National Health Service (NHS) invested over £100 million between 2020 and 2022 and developed a network of 90 specialist clinics across England [9, 10]. The development of services was carried out at a pace with limited national guidance or research, and adapting to local contexts resulted in heterogeneous delivery, and no standardised best practice model was identified [11, 12, 13].

Care for this complex condition took on a treatable traits approach [14], with self‐management as the longer‐term strategy after discharge from rehabilitation services [9]. Recent studies advocate for a standard of care to include a multidisciplinary approach to the assessment and management of LC and suggest that these should be both contextually appropriate and tailored to the symptoms [15, 16, 17, 18].

For some, accessing LC services and navigating the healthcare system has been reported as challenging [19, 20, 21]. Qualitative evaluation recommends better coordination of care across multiple pathways with care delivered as a person‐centred, holistic approach, incorporating listening and validation [21, 22, 23, 24, 25]. Quantitative monitoring of LC clinics provides insights into their clinical effectiveness. This has highlighted that, for many, symptoms persist for many years [26]; therefore, services need to support the long‐term impacts of LC and associated complexities [20, 25].

In 2024/5 the commissioning of LC services was transitioned to integrated care boards (ICBs). While this saw a reduction in funding it also provides an opportunity for LC service providers to consider alternative care models and amplify the voices of those with lived experience in re‐design [17, 18, 27, 28].

As identified by Kennelley et al. 2023 [29], ‘As the world begins to recover from the COVID‐19 pandemic, we must promote the voices and prioritise the needs of those who continue to experience long‐term impacts of the virus in research, clinical, and personal practice’. This emphasises the need to include views of patients still symptomatic after many years and to explore their changing care needs [20]. Therefore, this study considers the longer‐term perspectives of patients attending three heterogeneous LC clinics.

### 1.1 Objectives

This study aimed to explore individuals' perspectives following attendance at an LC service to explore:

1. Ongoing symptoms, functional limitations and current vocational status.
2. Understanding of recovery trajectory and facilitators.
3. Patients' perceptions of the LC care pathway and identify unmet care needs.

## 2 Methods

### 2.1 Methodology

We utilised a cross‐sectional online survey, using a convenience non‐probability sampling method to explore LC patients' perspectives and experiences of LC clinics. Three LC clinics from the LOCOMOTION consortium each independently completed a service evaluation survey to inform local ongoing care needs. The study protocol for LOCOMOTION, with details of management, governance and patient involvement, has been published elsewhere [30]. Ethical approval and subsequent amendments were granted by Yorkshire and The Humber—Bradford Leeds, Research Ethics Committee (REC ref: 21/YH/0276).

### 2.2 Patient and Public Involvement

The service evaluation survey was co‐produced with Experts by Experience who had previously attended an LC service, some of whom were members of the LOCOMOTION Patient Advisory Group. Collaboration and involvement continued throughout the study including analysis, interpretation and writing processes.

### 2.3 Participant

#### 2.3.1 Setting

LC services varied in set‐up and delivery. See Table 1 for summaries of clinics.

**Table 1:** Description of long Covid service site structure using a tiered classification (as previously described elsewhere [12, 31]).

Site	Description
Site A	Tier 3 clinic led by a respiratory consultant with a small multidisciplinary team (MDT) (psychologist, occupational therapist and physiotherapist). Primarily assessment service with referral for specialist input. Links with community‐based rehabilitation service.
Site B	Hospital‐based tier 3 clinic run by a respiratory physician and clinical research fellow. No formal MDT support, but can refer to the community rehabilitation team.
Site C	Entirely virtual community‐based tier 2 clinic jointly led by a general practitioner (GP) and an occupational therapist, and with a large MDT of allied professionals. Complex patients are reviewed by the GP and referred on to secondary care specialties as needed.

#### 2.3.2 Sampling and Recruitment

An invitation to take part in the service evaluation was sent to patients who had attended the clinic between August 2020 and May 2024. This represents approximately between 15% and 30% of each site's clinic attendance (data not shown). Invites were sent via email or text, including a link to the online survey (see Supporting File 1 and 2—Additional survey details and Online Survey). The survey was available online in Microsoft Forms and was available to complete between November 2023 and May 2024. The survey was only available in English.

### 2.4 Survey Components

#### 2.4.1 Development of Survey

The self‐report survey was designed by the multidisciplinary LC team at Site A. Patients were involved in survey design, including question development, survey content, structure and language. The survey was tested on 30 patients over the telephone and amended according to patient and clinician feedback. The survey included the following key areas: Functional impact and symptoms; Recovery and trajectory; Local healthcare support and unmet needs; and Vocational status and additional comments. It included a 3‐point Global Rate of Change (GRoC) scale [32] and the Post‐Covid Functional Status (PCFS) [33]. The PCFS scale includes a Likert scale (0–4) measuring symptom severity and functional limitations, where 0 is no symptoms, 1 negligible limitations (can perform all usual duties/activities, but with some symptoms), 2 slight limitations (occasionally needing to avoid or reduce usual duties), 3 moderate limitations (unable to perform all usual duties or activities including work) and 4 severe limitations, (being dependent on nursing care or assistance from another). See Supporting Files 1 and 2—Additional survey details and online survey, for further information.

### 2.5 Data Collection

Participants were given a pseudonymised ID number to use when completing the survey; no personal identifiable data was requested or recorded. Health‐related data were captured from local trust databases and/or clinical notes. Demographic data were taken directly from available data stored on Trust databases. Information was stored at each local site in accordance with the trusts' information governance policies.

### 2.6 Data Management

Survey responses were stored locally to permit analysis, and de‐identified data were shared between the three sites using secure online SharePoint. All sites adhered to local governance procedures and were compliant with the study's ethical approval.

### 2.7 Analysis

The survey recorded all data digitally on the Microsoft (MS) Forms platform. All data were exported to MS Excel and additional demographics data were added by each site to create the full dataset. Quantitative data were analysed using MS Excel to synthesise descriptive analysis, frequency distribution, percentages and measures of dispersion. Questionnaire and demographics data were cross‐tabulated to investigate relationships between multiple variables in line with our aims.

Qualitative data from the free‐text sections of the questionnaire consisted of short sentences and paragraphs, which were analysed using thematic analysis following Braun and Clarkes approach [34] adapted for brief written responses [35]. Researchers from each site (P.W., C.L., and S.J.) conducted line‐by‐line inductive coding independently to ensure codes were grounded in the data. Coding was informed by the research objectives—to explore the impact LC continues to have on patients, their perceptions of their recovery, the LC care pathway, care provided, and ongoing care needs. The local evaluators discussed and compared initial codes to develop an agreed‐upon coding framework. Codes were grouped into higher‐order conceptual concepts from which themes and sub‐themes were subsequently identified. This process was iterative and reflexive, where discrepancies were resolved through discussion with the wider team to enhance rigour. Themes were identified through an active interpretive process that allowed the team to recognise common and recurrent patterns in the data, while remaining attentive to variations across sites. This supported a rich and contextualised understanding of patients' experiences and perceptions of their LC service (see Supporting File 3—Qualitative Analysis) [36].

**Figure 1:**
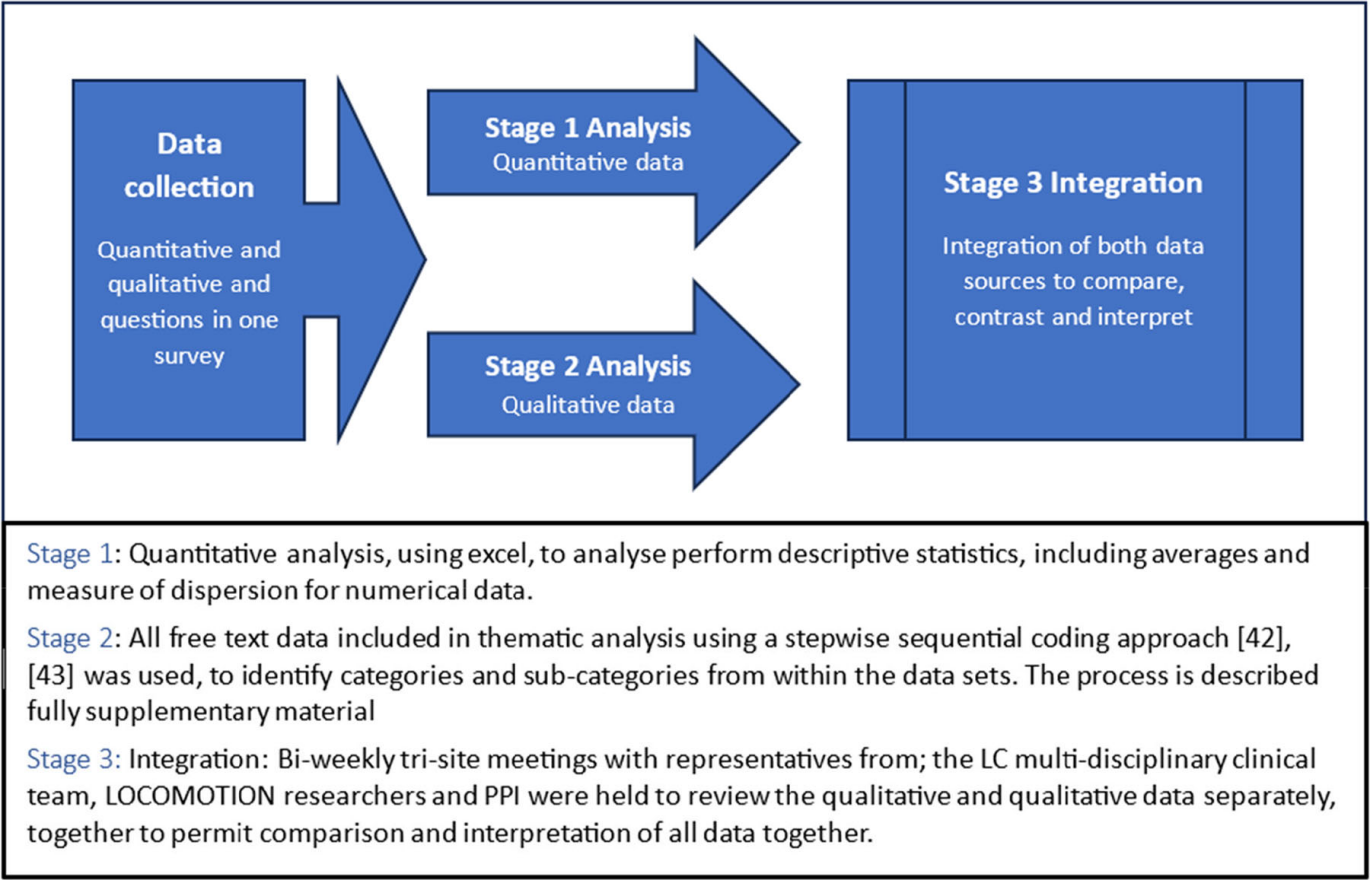
Illustration demonstrating the mixed‐methods convergent parallel design approach employed for analysis.

Data from the three sites were combined and analysed in conjunction with a mixed‐methods convergent parallel design principle permitting numerical data to be situated in the context of qualitative data [37], see Figure 1 for details.

## 3 Results

### 3.1 Participants

Of the 513 patients who were invited, 269 (52%) responded, of which 30 (11%) completed it via telephone with a healthcare professional. Similar participant characteristics, response rate and survey results were observed across all three sites. Response rates for each site were A = 52.4%, B = 61.5% and C = 47.3%, with a mean of 52.4%. Data were combined and analysed as one dataset with no between‐site analysis.

Demographic data for both responders and non‐responders are listed in Table 2. Responders had a mean age of 52.7 (± 12.0); it was noted that under 40‐year‐olds responded less frequently (*n* = 38, 14.1% vs. *n* = 61, 25.0%). The sample was predominantly female 69.1% (*n* = 186) and white‐British 55.0% (*n* = 148). Indices of multiple deprivation (IMDs) saw some differences between responders and non‐responders. While similar in the lower two quintiles (1 and 2), IMD groups 3 and 4 saw more non‐responders, compared to an elevated response rate in those least deprived (IMD 5) (*n* = 79, 29.9% vs. *n* = 47, 19.3%). This may reflect access to clinics, as the service evaluation was not designed to explore predictions of participation. We did not analyse this further.

**Table 2:** Characteristics of participants with comparison of responders and non‐responders.

	Responders		Non‐responder		All	
	*N* = 269	% or SD	*n* = 244	% or SD	*n* = 513	% or SD
Age						
< 30	10	3.7%	20	8.2%	30	5.8%
30–39	28	10.4%	41	16.8%	69	13.5%
40–49	61	22.7%	63	25.8%	124	24.2%
50–59	93	34.6%	64	26.2%	157	30.6%
60–69	52	19.3%	37	15.2%	89	17.3%
> 70	22	8.2%	19	7.8%	41	8.0%
Missing	3	1.1%	0	0.0%	3	0.6%
Mean age	52.7	12.0 (SD)	49.4	13.6 (SD)	51.1	12.9 (SD)
Sex						
Sex (female)	186	69.1%	178	73.0%	364	71.0%
Sex (male)	80	29.7%	66	27.0%	146	28.5%
Missing	3	1.1%	0	0.0%	3	0.6%
Ethnicity						
White—British	148	55.0%	128	52.5%	276	53.8%
White—Any other White background	34	12.6%	29	11.9%	63	12.3%
Asian—Asian British (including British Indian/Pakistani)	3	1.1%	7	2.9%	10	1.9%
Asian—Any Other Asian Background	19	7.1%	17	7.0%	36	7.0%
Black or Black British—African/Caribbean	9	3.3%	12	4.9%	21	4.1%
Mixed—Any other mixed background	4	1.5%	4	1.6%	8	1.6%
Other Ethnic Groups—Any other ethnic group	37	13.8%	34	13.9%	71	13.0%
Missing	15	5.6%	13	5.3%	28	5.5%
Total	269	100.0%	244	100.0%	513	100.0%
Index of multiple deprivation—(Quintiles)						
1	24	8.9%	27	11.1%	51	9.9%
2	42	15.6%	34	13.9%	76	14.8%
3	48	17.8%	63	25.8%	111	21.6%
4	54	20.1%	60	24.6%	114	22.2%
5	79	29.4%	47	19.3%	126	24.6%
Missing	22	8.2%	13	5.3%	35	6.8%
Total	269	100.0%	244	100.0%	513	100.0%
Predominant UK variant at time of infection						
Pre‐Alpha	113	42.0%	111	45.5%	224	43.7%
Alpha	66	24.5%	46	18.9%	112	21.8%
Delta	25	9.3%	21	8.6%	46	9.0%
Omicron	25	9.3%	10	4.1%	35	6.8%
Missing	40	14.9%	56	23.0%	96	18.7%
Total	269	100.0%	244	100.0%	513	100.0%
Mean duration of LC (known only)	1204.4	275.7 (SD)	1262.1	252.6 (SD)	1230.5	266.8 (SD)
**Severity of initial infection (*n* = 306) only available for two sites						
Community	144	84.2%	118	87.4%	262	85.6%
Hospital‐Ward	18	10.5%	13	9.6%	31	10.1%
Hospital‐ITU	4	2.3%	2	1.5%	6	2.0%
Vaccine triggered	1	0.6%	1	0.7%	2	0.7%
Missing	4	2.3%	1	0.7%	5	1.6%
Total	171	100.0%	135	100.0%	306	100.0%
Duration of long Covid						
< 1 year	1	0.4%	0	0.0%	1	0.2%
1–2 years	16	5.9%	8	3.3%	24	4.7%
2–3 years	49	18.2%	34	13.9%	83	16.2%
3–4 years	106	39.4%	89	36.5%	195	38.0%
> 4 years	59	21.9%	60	24.6%	119	23.2%
Missing	38	14.1%	53	21.7%	91	17.7%
Total	269	100.00%	244	100.00%	513	100.0%
Interval since first assessment at the LC clinic						
< 1 year	105	39.0%	108	44.3%	213	41.5%
1–2 years	87	32.3%	54	22.1%	141	27.5%
2–3 years	32	11.9%	22	9.0%	54	10.5%
> 3 years	7	2.6%	7	2.9%	14	2.7%
Missing	38	14.1%	53	21.7%	91	17.7%
Total	269	100.0%	244	100.0%	513	100.0%
Long Covid service						
Site A	99	36.8%	90	36.9%	189	36.8%
Site B	72	26.8%	45	18.4%	117	22.8%
Site C	98	36.4%	109	44.7%	207	40.4%
Total	269	100.0%	244	100.0%	513	100.0%

* Data is only known for two sites; the third reported a similar dominance of community‐managed infections.

** English IMD for 2019 and Welsh IMD for 2019 were used.

Duration of LC was on average ≥ 3 years (mean 1204.4 days, ± 275.7). Duration between survey completion and first contact with the LC specialist team was largely within the previous 1 (39.0%, *n* = 105) or 2 (32.3%, *n* = 87) years. The initial infection variant period for responders was mainly pre‐Alpha (42.0%) or Alpha (24.5%), and most acute infections were managed in the non‐hospital setting (84.2%, *n* = 144 out of 301 for two sites). We had a high rate of missing data regarding infection details, and one site did not provide the severity of infection, so it was omitted from this category.

### 3.2 Stage 1: Quantitative Analysis

Data are presented as counts, percentages, and measures of dispersion. Responders answered questions based on the response to the previous question; therefore, the denominator value varies between questions. For survey results, the denominator includes pre‐defined category responses and excludes ‘other—free text’ or unanswered ‘missing’ data. The exception is where free text, via explicit wording or content, strongly aligned with the categories. Where there was agreement by two researchers, it was re‐coded as quantitative count data. The free‐text data remained available for qualitative analysis and coded into themes in Stage 2 of the analyses.

#### 3.2.1 Current Functional Impact and Symptoms (Questions 2, 3, 4, 17 Functional Impact, Symptoms and Vocational Status)

##### 3.2.1.1 Functional Status

The PCFS scale identified that most participants had not fully recovered (253 out of 269, 94.1%) with differing levels of functional impact: negligible (10.0%), mild (28.3%), moderate (42.0%) or severe (13.8%) (see Supporting File 4 Table A1). Note, when comparing demographics across the PCFS levels, some counts are small (*n* ≤ 5), particularly ethnicity and IMD, and therefore should be interpreted with caution.

##### 3.2.1.2 Symptoms

**Figure 2:**
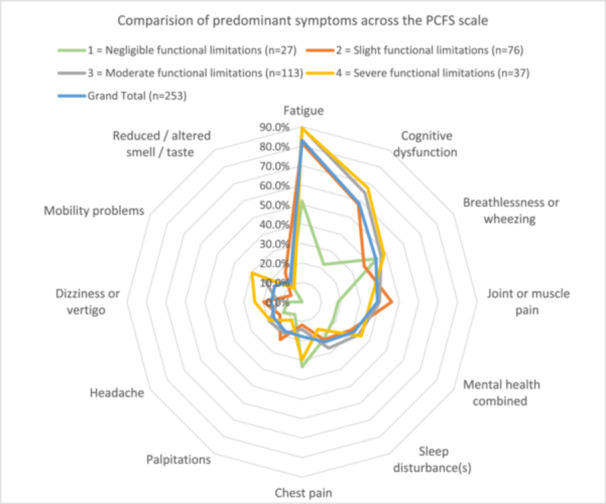
Radar chart comparing the most frequently reported predominant symptoms across different PCFS levels.

The 253 responders, indicating they had not fully recovered, reported their top five most frequently experienced symptoms as fatigue 83.0% (*n* = 210); cognitive dysfunction 58.5% (*n* = 148); breathlessness or wheezing 43.9% (*n* = 111); joint/muscle pain 38.7% (*n* = 98) and mental health impact 30.8% (*n* = 78). For mental health, we combined anxiety (*n* = 44) and depression (*n* = 46) into one category, where 12 participants indicated both, and therefore, were only counted once. Figure 2 shows the percentage of reported symptoms by PCFS (see Supporting File 4—Figure A4 for bar chart presentation). This highlights that symptom groups are comparable across the PCFS mild/moderate/severe scale.

##### 3.2.1.3 Vocational Impact

**Figure 3:**
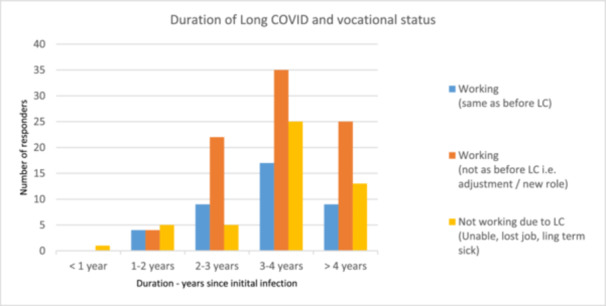
Duration of LC symptoms according to vocational status.

Vocational status was provided by 85.9% (*n* = 231). Data were analysed and grouped into four categories: (1) ‘working—but not the same as before LC’, that is, with adjustment, reduced hours, change in duties or role, or lost job but since found a new role, 42.0% (*n* = 97); (2) ‘unable to work’ either on long‐term sick, lost job or unable to work, 25.5% (*n* = 59); (3) ‘Working—as prior to their initial LC infection’, 20.8% (*n* = 48); and (4) ‘N/A—not working prior to LC’, 11.7% (*n* = 27). Duration of LC was cross‐tabulated with vocational status, see Figure 3. The majority with LC beyond 2 years reported ‘working—but not the same as before LC’ as the most frequent, and beyond 3 years ‘working as prior’ was the least reported status.

##### 3.2.1.4 Trajectory of Recovery and Faciliatory Factors (Questions 5, 6 and 7)

Using a GRoC scale with a simple 3‐point scale [32], it was possible to capture participants' previous 6‐month recovery. There were 246 who responded, 47% (*n* = 100) were at a plateau, 39.0% (*n* = 96) were making progress, and 20.3% (*n* = 50) were worse. Those with a more limited PCFS score (moderate and severe) reported a worse trajectory, but recovery was relatively consistent regardless of initial assessment date (see Supporting Information 4—Additional tables and figures). Further insight from those reporting recovery identified provision of information and advice from the LC clinic (40.6%, *n* = 41), medication (22.8%, *n* = 23) and onward referrals (16.8%, *n* = 17) as aiding recovery.

#### 3.2.2 LC Healthcare Provision and Ongoing Care Needs (Questions 13, 14, 15 and 16)

##### 3.2.2.1 Current Provision of Care/Support

Current access to care/support was reported by 245 participants. Over half, 56.7% (*n* = 139), reported they were not receiving care despite being symptomatic, 33.0% (*n* = 81) were currently receiving care/support and 10.2% (*n* = 25) identified ongoing care as not necessary. On a follow‐up question, 95.0% (*n* = 77) provided further details about the provision of care (they could select more than one option). Healthcare support was provided by the NHS (56.6%, *n* = 73), private/alternative (29.5%, *n* = 38) and others (4.5%, *n* = 7), such as the ENO breathe programme [38] or participating in LC research (8.5%, *n* = 11).

**Figure 4:**
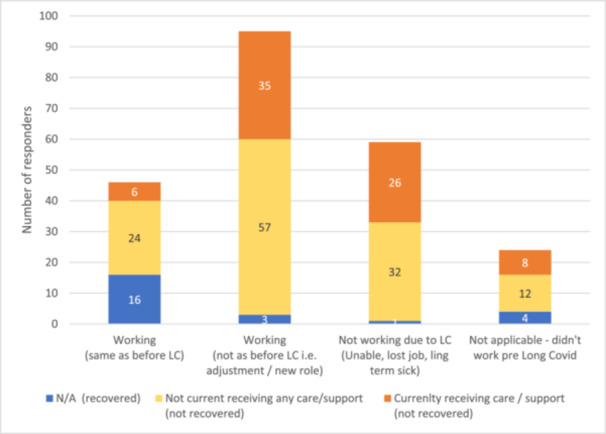
Self‐reported identification of current care provision by vocational status category.

For those who had not recovered, vocational status was cross tabulated with current care provision. Out of 200, 150 reported they were not working in the same capacity as before Covid‐19 infection (either not working or working with adjustments). Of these 150, 40.7% (*n* = 61) were receiving care/support, and over half, 59.3% (*n* = 89), were not (see Figure 4).

#### 3.2.3 Perceived Ongoing Care/Support Needs

All who had ‘not fully recovered’ were asked about additional care/support needs. There were 201 who responded, 62.7% (*n* = 126) additional care (including 36 who were receiving some care/support). 194 answered both questions (receiving care and additional care needs), which revealed four groups: (1) Receiving care—no additional care needed, (2) Receiving care—identified additional care is needed, (3) Not receiving care—identified care is needed and (4) Not receiving care—no support/care needed.

**Figure 5:**
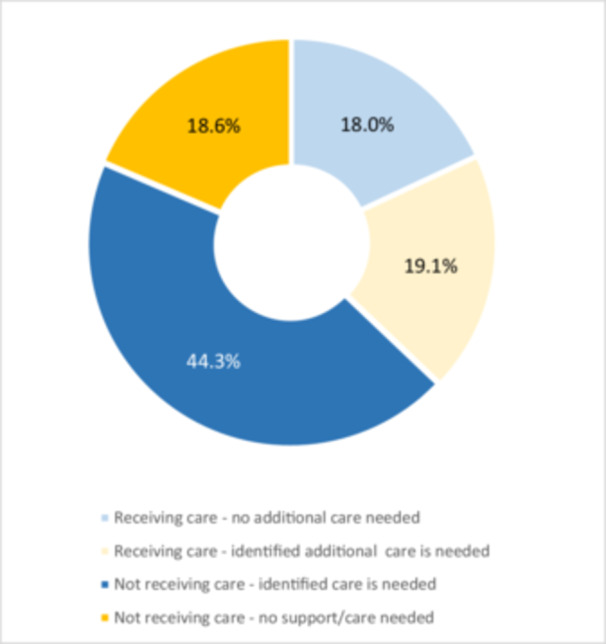
Self‐reported identification of current care provision and perceived ongoing care needs.

**Figure 6:**
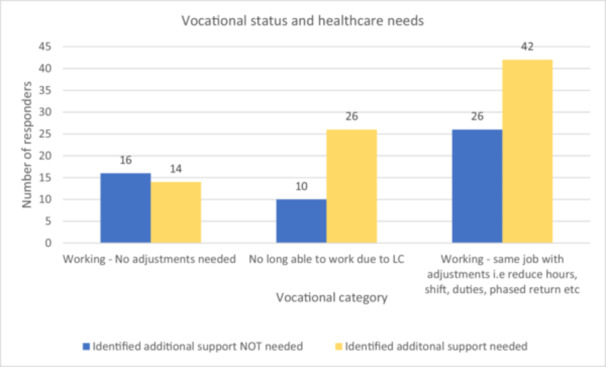
Self‐reported identification of additional healthcare needs by different vocational status.

44.3% (*n* = 86) had not fully recovered and had unmet care needs, while the other groups ranged between 18.1% and 19.2% (see Figure 5. Participants' current and perceived ongoing care needs). Furthermore, those who were ‘no longer working’ (*n* = 26 out of 36) or ‘working with adjustments’ (*n* = 42 out of 68) reported a high frequency of unmet care needs, compared to those working in the same capacity (*n* = 14 out of 30) (see Figure 6).

Overall, perceived unmet care needs were noted to be slightly greater in those who were younger (mean age 50.4 ± 11.6, vs. 54.6 ± 11.6), had a longer duration of LC (mean 1223 ± 263 vs. 1196 ± 263), and had their initial infection treated in the community. Low numbers in ethnicity and IMD categories with missing data make extrapolation of findings for diversity and inclusion difficult. Further analysis to explore the statistical difference was not performed, as we did not design the study to explore this.

### 3.3 Stage 2: Qualitative Thematic Analysis

**Figure 7:**
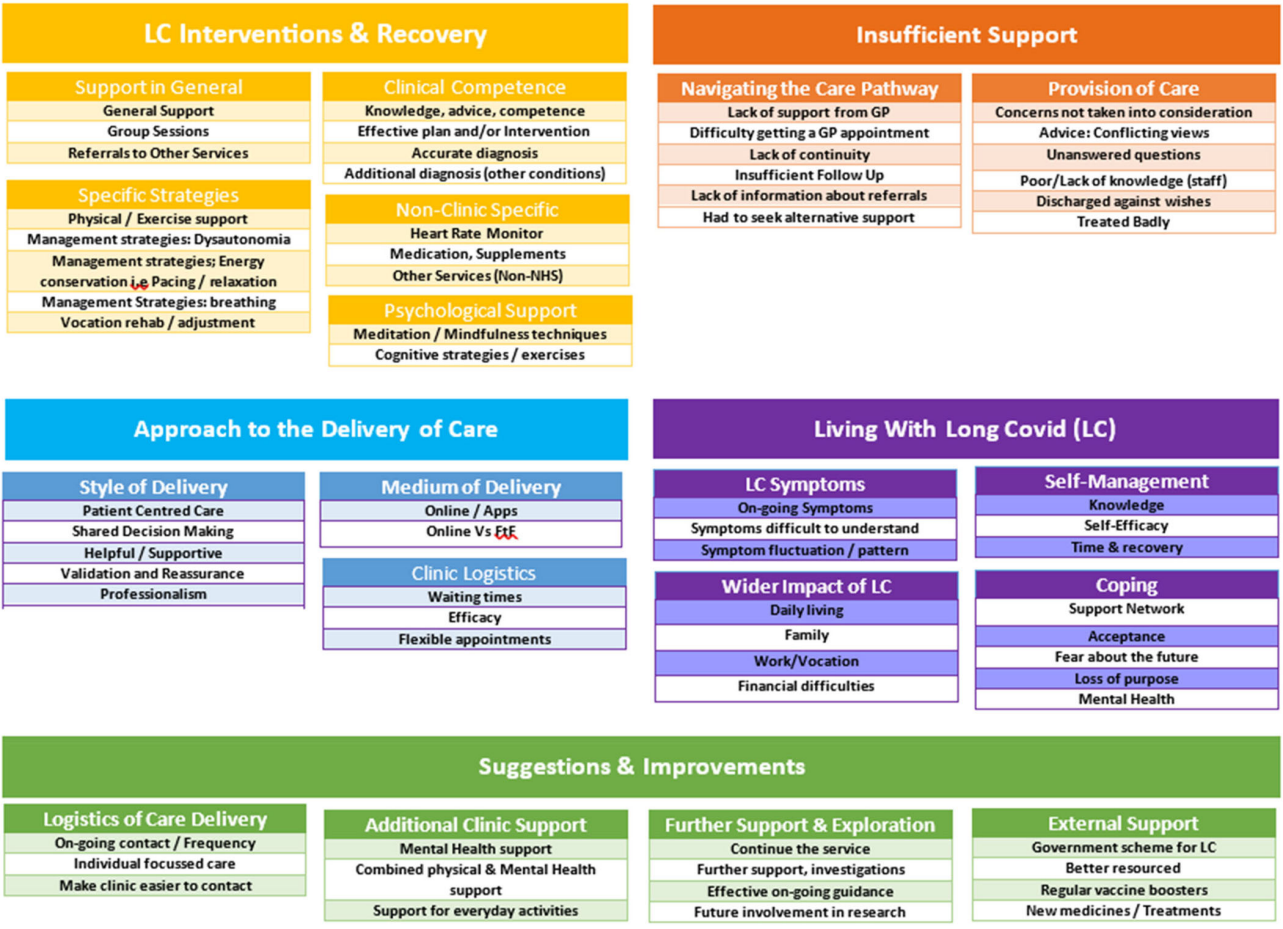
Qualitative analysis: Emergent domains, themes and sub‐themes.

Thematic analysis of the qualitative data identified five overarching domains. Contained within the domains were themes and sub‐themes identified by the similarity and recurring nature of text supplied by participants (see Figure 7). The five domains were as follows:

- LC Interventions and recovery (5 themes; 17 sub‐themes).
- Approach to the delivery of care (3 themes, 10 sub‐themes).
- Insufficient support (2 themes, 12 sub‐themes).
- Living with LC (4 themes, 15 sub‐themes).
- Suggestions and improvements (4 themes, 14 sub‐themes).

The overarching domain, ‘LC Interventions & Recovery’, encompassed all themes related to patients' perceptions about the support provided by LC clinics, including the care pathway and subsequent clinic referrals. Unlike the domain ‘LC Interventions & Recovery’, the overarching domain ‘Approach to the Delivery of Care’ captures participants' perceptions relating to the way in which care pathways and referrals were delivered. Participants indicated they experienced a level of ‘Insufficient support’. This domain was constituted by two themes relating to navigating the care pathway and those related to the provision of care. The domain ‘Living With Long COVID’ involved both the immediate impact of LC (i.e., symptoms and coping with such symptoms) and the wider impact (i.e., on family life, activities for daily living, ability to work and financial implications). Finally, ‘Suggestions & Improvements’ focused on participants' suggestions for service improvement.

### 3.4 Stage 3: Integrated Findings

The mixed‐methods approach enabled quantitative data to be enriched by adding the patients' experiences captured by the qualitative component.

1. What impact does LC continue to have on patients who have attended the LC Clinic, including symptoms, functional limitation and vocational status?
  Fatigue, breathlessness, cognitive dysfunction, chest pain and a negative impact on mental health were the prevalent ongoing symptoms of LC as demonstrated by the quantitative data. The domain ‘Living with Long COVID’ involved aspects of participants' experiences in relation to LC symptoms, such as fluctuation and unpredictability, as well as the ongoing nature of the condition, which is difficult to understand. Further, nearly all (94.1%) responders identified ongoing symptoms as having an impact on daily function. This was expressed in the domain ‘wider impact of LC’, where it was reported that LC impacted personal life, activities for daily living such as housework, and difficulties with family life due to fatigue.
  From the quantitative analysis, it was evident that LC was experienced as having a significant impact on work and vocation status, for example, only 20.8% of participants were currently working in the capacity they had held before LC. This was echoed in the participant narratives, with accounts of making concessions for work or stopping work altogether due to LC. Finally, the culmination of reducing or stopping work had inevitable financial and psychological implications for some.
2. What is the trajectory of patient recovery, to what extent do patients believe they have recovered from LC, and what aspect of care facilitated recovery?
  Quantitative analysis indicated aspects of LC services and healthcare support that aided recovery, such as the provision of information and advice (40.6%), medication (22.8%) and onward referrals (16.8%). The domain, ‘LC Interventions & Recovery’, encompassed participants' experiences of support provided by LC clinics. Noteworthy aspects that patients reported as aiding recovery included LC group programmes, energy management principles, breathing exercises and psychological support, as well as support found outside the healthcare system, such as non‐NHS services and medication and supplements (Table 3).
  Quantitative analyses indicated that participants reported LC recovery as a non‐linear process, 39.0% identifying an upward trajectory within the 6 months before completing the survey and 20.3% indicating worsening symptoms.
  The domain ‘Living with LC’ indicated that participants perceived LC clinics as providing a general role in recovery as well as bespoke interventions provided by LC clinic specialists. The way in which such services were perceived was, in part, related to the knowledge and competence of clinical staff and practitioners. This was reflected in the emphasis placed upon receiving an accurate diagnosis of LC. Non‐clinic themes (e.g., non‐NHS services) were also indicated as playing an important role in aiding recovery.
  The domain ‘Coping’ involved important perceptions related to the role of support networks such as family, friends and peers. The importance of being self‐resolving in identifying and employing strategies to manage living with LC was also a strong theme. Some illustrated an acceptance that it had become their ‘new normal’. However, there were many participants who indicated the condition had a negative impact on their mental health and expressed challenges in relation to living with a condition that is unpredictable, leading to fears about the future. Some also reported that LC had an impact on their self‐perception and that they had experienced an overall loss of purpose.
3. How do patients perceive the LC care pathway, care received and ongoing care needs?
  **Table 3:** Thematic analysis results of free text survey comments, presented as domains, themes, sub‐themes and illustrative quotes.

Themes	Sub‐themes	Illustrative quotes
Domain: LC Clinic and Recovery		
Support in general	General support	‘Support from the COVID Rehab service. Access to my GP for help was not available so I found the service really helpful, as I often could not understand my symptoms’ (C12)
	Group sessions	‘I was supported from the beginning of the long covid clinics being set up for a number of months. The support groups and the medical care was such a help throughout’ (C70)
	Referrals to other services	‘Support, advice, knowledge and referrals to the right specialist with symptoms that have happened or been made worse by long Covid’ (C43)
Specific strategies	Physical/exercise support	‘I did have relapses but they got less severe and further apart. The pacing, particularly of taking up exercise again, did help but I still don't feel I have regained my pre‐Covid fitness levels, although I'm still working on it’ (C84)
	Management strategies: Dysautonomia	‘Received advice and support for pots type symptoms, 48 h ecg and bloods. Given support and information including the WNO wellness’ (B11)
	Management strategies: Energy conservation, that is, pacing/relaxation	‘The pacing I found invaluable as did ruling out any other reasons for my ongoing symptoms.’ (C5)
	Management strategies: Breathing	‘Recovery group 360 mind body & soul—peer support, mind‐body approach, breathwork and yoga’ (A455)
	Vocation rehab/adjustment	‘Going back to work successfully and understanding fully that my body is more or less fully recovered and functioning well’ (A988)
Psychological support	Meditation/Mindfulness techniques	‘Meditation and breath work to support my autonomic nervous system and severe anxiety and overwhelm’ (B42)
	Cognitive strategies/exercises	‘Psychologist has helped me and have bad dreams. XXXX has been very good with my progress’ (A1513)
Clinical competence	Knowledge, advice and competence	‘I believe the help given to me was showing a good understanding of all I was going through, the advice really helped and also it was good to know that I was listened to and knowing that the Dr was thorough in their knowledge and understanding of how Covid were truly affecting the patients of this illness’ (C47)
	Effective plan and/or Intervention	‘The overall plan was well put together and has made it possible for me to function in a way that I am able to work and live a relatively normal life’ (C52)
	Accurate diagnosis	‘I am most terribly grateful for all the care, attention and support I have received. When I first attended in March/April 2021 I had not up to then had a formal diagnosis of long covid, and felt that I had finally found a place of safety, somewhere that knew what they were doing and would look after me, and you did’ (B26)
	Additional diagnosis (other conditions)	(The LC clinic) … ‘enabled me to get a diagnosis of autonomic dysfunction. Exclude POTs and start medication to help me get back to work’ (C21)
Non‐clinic specific	Heart rate/sleep monitor	‘Wearing a Fitbit to monitor exertion and sleep’ (C96)
	Medication and supplements	‘My GP has suggested various non‐medical tablets: NADH, NAC+, MAGNESIUM FLYCINATE, COQ‐10, GLUTATGIONE, PQQ, AND NAC’ (C71)
	Other services (Non‐NHS)	‘I wish there was a focused tai chi or similar group, with an opportunity to meet others, like the WNO programme (but with some social interaction too). Other groups have regular meet ups to give information for the first part like a speaker, or updates on research, techniques to learn then have a general chat session. It can be face to face, on line or hybrid’ (B16)
Approach to the Delivery of Care		
Style of delivery	Patient‐centred care	‘Support and communication from Llandough and the Long Covid Rehab team was excellent, especially considering this was the early days of recognising Long Covid. Communication from both teams was also excellent. If I needed information you responded promptly and this was really important to my wellbeing at the time’ (B58)
	Shared decision‐making	‘The whole process was a two way dialogue, in which I was able to share my journey of recovery in the hope of helping others and l was able to ask about others and how they were affected. This was very important to me in terms of trying to understand my illness and what was happening’ (A988)
	Helpful/supportive	‘I honestly wasn't expecting much of a follow up but I saw a physio for the breathlessness. She was helpful, offering breathing exercises and answering my concerns’ (C12)
	Validation and reassurance	‘Long Covid Clinic has provided invaluable support. Validation and assistance with my symptoms has made a great difference to me’ (B11)
	Professionalism	‘Thorough examination which identified how long covid was affecting my body. Professional advice, when other professionals had limited knowledge on the subject, help and support at a very difficult time’ (B71)
	Holistic approach	‘I think your service was invaluable it actually looking at me as a whole person and breaking down all the blood tests and realising what was happening to me regards lack of ferritin, b12 and vitamin D and other symptoms and getting appropriate treatment’ (C47)
Medium of delivery	Online/Apps	‘B12 Zoe app, self‐treatment and research’ (B77) ‘psychologist, he helped but no referral, downloaded a few apps on the phone but feel my issues remain the same’ (A110)
	Online versus FtF	‘Once I eventually got referred, the Covid Rehab team where quick to put in place the referrals for me. Although I did not have any Face to Face appointments, that the webinars I attended online where useful and informative, it was great to hear advice and the stories of other Long Covid sufferers. The online and telephone support of a counsellor help also’ (C22) ‘Having some face to face meetings would be beneficial however i do appreciate at the time it was not always possible’ (C52)
	Waiting times	‘Good response and adequate waiting time, appointment times were flexible and achievable’ (C23) ‘It took a while to be referred to the Long Covid Clinic but once picked up I was given a plan very quickly’ (C43)
Clinic logistics	Efficacy	‘I was very lucky in that I happened to ring the doctors on the day the service opened so I was seen, tested and assessed quickly’ (H84) ‘Everything was carried out quickly and I was referred to the appropriate healthcare team to help me manage my long covid’ (B85)
	Flexible appointments	‘Good response and adequate waiting time, appointment times were flexible and achievable’ (C23)
Insufficient Support		
Navigating the care pathway	Lack of support from GP	‘All the referrals and the way it was dealt was great just the after support from my own GP surgery had been poor as they was almost unable to understand my needs’ (B80)
	Difficulty getting a GP appointment	‘No they were cancelled and I was not informed. Go back to my GP … you try and get an appointment!’ (C76)
	Lack of continuity	‘The local G.P was not aware of my being part of a trial Long Covid scheme and was dismissive and or unknowledgeable … so much so, it took me to move practices in order to review my meds which Id been kept on since July 2023 which under my new G.P have been weened off. In answer to this question, probably a line of communication between the Trial/referral team to my local G.P at that time’ (B104)
	Insufficient follow‐up	‘I went to a long covid clinic once. Was assessed by four practitioners and was then sent a letter confirming i had long covid and that was it. No treatment. No referrals. No support. Nothing whatsoever’ (A532)
	Lack of information about referrals	‘It was all very stressful and didn't know what I had been referred for as not everything was in the email’ (C29)
	Had to seek alternative support	‘I have been awaiting PCAC service at UCLH to see a particular doctor—I paid to see someone privately [name], and am unable to work for 3 yrs and I got in touch with my private doctor and asked me to see him via NHS but has not been able to see him. I have been asked to be referred again and test has been done but has not been able to seen in the last 4 months’ (A594)
Provision of care	Concerns not taken into consideration	‘I felt my concerns were not always taken into account…. I advised that my other health conditions were an issue and that my other conditions were made worse’ (C31) ‘I just feel my breathing problems have not been fully investigated’ (B102)
	Advice: Conflicting views	‘Given lots of conflicting advise from different specialist and has not made any material difference’ (A594)
	Unanswered questions	‘I have no answers to what I should or shouldn't be doing. Just been left to get on with life’ (A408)
	Poor/Lack of knowledge (staff)	‘It felt like there was a complete lack of understanding of post viral illness and the enormity of the impact it can have on life but that with good support, improvement is possible’ (A644)
	Discharged against wishes	‘The pacing I found invaluable as did ruling out any other reasons for my ongoing symptoms. Although, like many others I was discharged against my wishes from the service whilst still ill and left to fend for myself which was/is very disappointing’ (C5)
	Treated badly	‘I genuinely feel like I am invisible to the world and to the NHS. I have had to spend the last two years finding a way to survive on my own without help. I have had numerous referrals and each and every time I am told they have no solid answers for this’ (A1041)
Living With Long Covid (LC)		
LC symptoms	Ongoing symptoms	‘I am 4 years in now and still struggling to hold down my job as a teacher. Because I am managing lots of my symptoms through pacing and I know how to look after myself with diet and exercise etc., I feel I've just been left to get on with things, yet it would appear I have a long term chronic condition. I am not aware if treatments have been found that might help me, or tests to try and find out why I'm still suffering. In particular I want to try and find out whether the neurological issues are likely to be permanent or if there is anything I can do to help that side of things. I don't know how to access support with that’ (B58)
	Symptoms difficult to understand	‘The above list didn't mention my spontaneous allergic reactions, mast cell problems and I'm left in limbo generally without an immunologist who “believes” in mcas (last one told me he didn't believe in Ehlers‐Danlos Syndrome or MCAS then discharged me‐not very useful)’ (B16)
	Symptom fluctuation/pattern	‘A timeframe of continued support, whether support would be ongoing and how often to expect contact would be beneficial due to the ongoing and changing symptoms of lc’ (B11)
	Mental health	‘One thing I don't think has been fully taken notice of is my mental health and I feel I have PTSD from catching covid19 in work, having colleagues die, bringing covid home to my family, lack of appropriate PPE and loosing my career. All because NHS didn't fully protect or help me. I absolutely hate going near hospitals now. I haven't once seen or been referred to a psychiatrist. I relive all these experiences everyday and I want it to stop’ (B5)
Wider Impact of LC	Daily living	‘I go to the office three days a week and that is basically my social life. No eating out, no point because I cannot taste or smell anything. Confidence destroyed with society because of the amount of times I have been pushed and barged around even though I have to use a walking stick to get around now’ (A1041)
	Family	‘Although I'm working 3 h 45 a day I am so exhausted I have to sleep when I get home. The exhaustion has not changed. It effects my family life as I have no energy’ (C69)
	Work/Vocation	‘I have only been able to get back to part time work as I'm self‐employed, home based and because we had savings to fall back on. My life and my family's life has completely changed’ (C69) ‘Reduced my hours on health grounds as Manager wouldn't support working from home’ (B85) ‘Recently LC has affected my work greatly, as a result of that I have been missing work quite often. I feel I only have certain amount of energy and when i have been used it up, I get very ill and need long time to recover’ (A9303)
	Financial difficulties	‘Having to stop work, though I now have little money to live on and lack any real purpose in life’ (C5)
Self‐management	Knowledge	‘Understanding what saps my energy most so I'm able to plan, prioritise and pace’ (C7) ‘Awareness/knowledge resulting in acceptance’ (B27)
	Self‐efficacy	‘My determination to help myself and take to act upon the advice of the Covid Rehab team and my Life Coach’ (C22)
	Time and recovery	‘Just got better over time’ (C83)
Coping	Support network	‘The support of my partner’ (C45) ‘I do not receive any support/care for Long Covid, apart from my family’ (B109) ‘The support given by the long covid multidisciplinary Team was helpful at the beginning of the illness’ (B13)
	Acceptance	‘I have adapted to my fatigue and memory problems, accepting my current condition as my new normal. I have managed to retain my old job, that was fortunately always home based, but need to work a longer day than paid for to allow for extra breaks and additional memory aids’ (C30)
	Fear about the future	‘I am really grateful for the support I have received, and continue to receive, from the Long COVID Rehab team, OT [occupational therapy] team and IAPT team. It took a long time to get to speak to people who could help and I have been very fortunate to have been seen faster than initial appointments were allocated thanks to interventions from the team. I know that the team is small, under‐resourced and overstretched. They have been so helpful and supportive. I would not be where I am without their support. I am trying to go back to work and hope I will succeed but am worried that I won't be able to and what will happen to me then’ (C60)
	Loss of purpose	‘Having to stop work, though I now have little money to live on and lack any real purpose in life’ (C5)
Suggestions and Improvements		
Logistics of care and delivery	Ongoing contact/frequency	‘I am being forced to retire from a job I loved in the nhs because of an illness I caught working for the nhs. I feel regular follow ups would help with the anxiety and depression and not make me feel it's all in my head and not believed’ (B9)
	Individual‐focused care	‘A recognised diagnosis of Long COVID with individualized support that takes account of symptoms’ (B36) ‘For the condition to be viewed holistically by healthcare providers, not having each body part looked at in isolation, and for the referrals to look at the specifics of the condition’ (A834)
	Make the clinic easier to contact	‘It would be good to have contact details of the Long Covid clinic’ (C71)
Additional clinic support	Mental health support	‘I would appreciate mental health support via the NHS, specialised in Long COVID. I have been diagnosed with “reactive depression” during these sessions, namely as a reaction to having now longstanding fatigue and not being able to do the things I was able to do before’ (A489)
	Combined physical and mental health support	‘I'm not sure if combined physical/mental health rehab might have helped me’ (C7)
	Support for everyday activities	‘My dad used to help me with lots of things like finances and forms, but he died suddenly in September. My reading is not always good, so things are hard. Maybe some support here?’ (C88)
Further support/exploration	Continue the service	‘continue the service and work needed to increase knowledge and ways of dealing with patients left to suffer with long covid’ (C45)
	Further support and investigations	‘But even if you don't have a solution for us, it would be nice if we could at least see that you are trying, by being actively involved in ongoing investigations/trials/updates/therapies and just general willingness to still be available to us as we still very much need help and support’ (A528)
	Effective ongoing guidance	‘Effective guidance on how to manage long covid’ (C57)
	Future involvement in research	‘I also would like to be involved in any potential research with medication or alternatives that could help. I am taking biologics injections (immunosuppression) and this worsens my fatigue. I would be willing to participate in any research in medication (or alternative treatments) that could potentially help to improve fatigue’ (A489)
External support	Government scheme for LC	‘Maybe some kind of government scheme that helps rehabilitate long covid sufferers’ (C14)
	Better resourced	‘I just wish the service was better resourced. However, that, sadly, applies to the whole NHS’ (C60) ‘I really appreciate your work but I feel you have not been given the financial support or resources to investigate fully or investigate the multi‐systemic nature of this’ (B16) ‘Incredibly grateful for the tireless efforts of Dr XXXX and team. often despite lack of dedicated funding and time, she has led the way in provision of care for LC patients (who are often despairing, exhausted and very much in need), as well as local research and links with others for research. Her expertise and constant hard work is without question. Better funding for dedicated service, which is sustainable, is needed’ (B31)
	Regular vaccine boosters	‘I'm strongly feel that those living with long covid ought to receive regular vaccine boosters. The last thing I want to happen is to catch COVID *again*. I dread this and it is a constant source of stress and worry. I fear I will get knocked back to the beginning and have a major fatigue relapse’ (C60) ‘I was urged to get the vaccine which I had a bad reaction to, then urged to have a booster which I had an even worse reaction to and made my condition very bad again’ (B42) ‘More research, referrals to covid vaccines (we do not automatically get them from the nhs even though we're at risk once we got long covid)’ (A1039)
	New medicines/treatments	‘We need medications to combat the enormous fatigue. Group chats don't cut it. I'd already worked out that zero exercise helped and radical pacing was the most effective. Could have done with a referral to a benefits agency as I was unable to work for over a year. I am still working much less and more slowly’ (C77) ‘It's unfortunate that I got Covid again approx 3.5 years later although being so careful, after being so ill with initial infection. I know that treatment was limited in the beginning. Could you please inform me if you have a greater variety of treatment options for Long Covid patients’ (A1019)
  The domain ‘Approach to the Delivery of Care’ captured participants' perceptions relating to the way in which the care pathway and referrals were delivered. The theme ‘style of delivery’ encompassed participants' perceptions of the way in which care was delivered by clinical staff. Participants indicated the importance of care being delivered in a professional and supportive manner, being listened to and validated, being patient‐centred and the importance of shared decision‐making.
  The mode by which care was delivered, or the ‘medium of delivery’, also emerged as an important theme. The medium by which care was delivered was clinic‐dependent, where some participants did not receive face‐to‐face (FtF) appointments. Participants expressed both positive and negative perceptions of online and FtF methods. ‘Clinic logistics’ related to the way in which LC clinics were run. Some participants perceived clinics to be effective from the initial point of contact, and the notion that patients could have some flexibility around their appointments was indicated as important with respect to being able to attend appointments. Some participants experienced waiting times positively; that is, waiting times were considered acceptable under the circumstances of a pandemic. However, for others, waiting times for clinic appointments were perceived as lengthy and unacceptable, and some reported having to seek help elsewhere.
  The notion of insufficient and a lack of ongoing care was apparent in both the qualitative and quantitative data. Only 33.0% reported they were currently receiving care, despite 84.1% describing mild, moderate or severe ongoing daily functional limitations. The qualitative analysis included the domain ‘Insufficient support’. Some participants, for example, found it difficult to access GP appointments, and whilst others felt their initial assessment was beneficial, they did not feel the same about onward referrals, which were reported as poor or having a lack of continuity. A small number of participants indicated they felt they had been discharged against their wishes, and some felt they had been treated badly.
  The domain ‘Suggestions and Improvements’ included themes and sub‐themes relating to participants' perceptions relating to ongoing care and what the LC service might look like going forward. Suggestions included that LC clinics include some method of ‘checking in’ with patients as they experienced long periods of time managing the condition without feedback. Similarly, making clinics easier to contact was indicated as being beneficial.
  Participants' perceptions of what additional services could look like as well as what emphasis could be placed on providing future comprehensive LC care included more mental health support as well as support for everyday activities. The theme ‘Further Support & Exploration’ included participants' desires for the service to be continued for guidance and investigations, as well as opportunities for involvement in future research.

## 4 Discussion

We conducted a mixed‐methods evaluation of three LC services across England and Wales. This involved a total of 269 participants who had attended one of the LC services within the previous 4 years. Participants completed an online survey that focused on current LC symptoms, recovery from LC and the role LC services play in recovery. Participants were predominantly female (69.1%) and white British (55.0%), with a mean age of 52.7 ± 12.0.

Quantitative data indicated that most participants had not fully recovered from LC, reporting ongoing mild (28.3%), moderate (42.0%) or severe (13.8%) limitations. Similar to previous research [3, 4], this study reports that the most frequently reported symptoms are fatigue (83.0%), cognitive dysfunction (58.5%), breathlessness or wheezing (43.9%), joint/muscle pain (38.7%), and anxiety and depression (30.8%). According to previous research, the clinical presentation of LC symptoms involves fluctuation and relapses over time [39, 40]. Similarly, participants who took part in this study indicated that LC recovery was a non‐linear process, with 20.3% indicating worsening or symptoms that had plateaued (40.7%) over the 6 months before completing the survey. Symptoms were reported as variable, unpredictable and difficult to understand.

It has previously been reported that approximately 25% of those suffering from LC have significant functional limitations [8]. In our study, 28.3% of participants reported limitations which were mild, 42.0% moderate and 13.8% severe. In relation to vocational impact, only 20.8% indicated they were working as they were before the initial Covid‐19 infection, with just under half (42.0%) not able to work in the same capacity as before LC. A quarter of participants who completed the survey indicated they were currently unable to work for reasons such as losing their job or long‐term sickness. Over half of the participants reported they were symptomatic but not receiving care. Supporting those with LC to maintain, or return to work, is an essential role for LC services from a clinical [41] and economic perspective [42]. We acknowledge this is a relatively small snapshot; nonetheless, it identifies the need for longer‐term vocational support.

The picture that emerges from these results is that participants who still experience symptoms, −61.5% (*n* = 150/244), describe ongoing recovery needs (e.g., report their condition as ongoing or worsening) and a negative impact on their ability to work, with almost half report they are not receiving care. This evaluation demonstrates the ongoing need for LC clinics to support patients with their recovery and the necessity for the provision of interventions to reduce symptoms with a view to reducing the wider negative impacts of LC, for example, family life and work.

In relation to LC services, research has previously indicated that patients experience barriers to accessing and navigating the healthcare system [19, 20, 22, 23, 43, 44]. It has also been recommended that the coordination of LC care needs to be improved [21]. It was evident from our data that barriers experienced by participants included a lack of information in relation to referrals, a lack of continuity and integration of health care services across the care pathway, and insufficient follow‐up appointments. Furthermore, participants reported waiting times as being too long and expressed frustration at the difficulty contacting their GP and booking appointments.

Previous studies have suggested that a person‐centred approach is necessary to support those with LC [21]. Participants included in this study reflected positively on a patient‐centred approach as well as shared decision‐making as being beneficial to recovery. Some participants also indicated they felt their concerns were not always taken into consideration, some had unanswered questions, and some believed they were treated, or discharged against their wishes.

This study had several limitations. It was cross‐sectional by design and therefore not able to draw on perceptions that may change over time. However, it adds value to previous literature, which has primarily focused on exploring the recovery experience of post‐hospital patients with shorter durations of LC [27, 28]. We explored the perspectives of patients with an average disease duration of over 3 years, where 21.9% had a duration of 4 years or more and 84.2% were treated in a community setting.

The survey utilised for the study is not a validated measure, limiting its generalisability; however [17, 18], public involvement was embedded in the design to ensure its development was guided by patient experience. Additionally, the data is presented as one cohort, and we recognise that services were heterogeneous, that is, the delivery of care, care pathways, staff and geography. We did not explore contextual nuances between sites [12, 21, 43].

Lastly, research has shown that those who decide to participate in online surveys are more likely to adhere to measures that can minimise the risk of being infected with SARS‐CoV‐2 [45]. Therefore, the relevance of these findings to the general population is limited, as it is likely that those who took part were more likely to adhere to treatments and interventions designed to improve LC, therefore, presenting a more positive picture than what perhaps exists in the general population. Despite this tendency for positivity, a significant number of respondents still felt their needs were not met by their LC service.

## 5 Conclusion

This evaluation adds to the body of LC literature through the exploration of patient perspectives from community‐based healthcare settings, including participants living with LC for extended durations. It examined the ongoing impact that LC has on patients' lives, the trajectory of their recovery and ongoing care needs, and patients' perspectives of the LC care pathway. Positive perceptions of the role LC services play in recovery, centred on the interventions for LC itself and the way in which care was delivered.

The findings highlight that LC services need to extend beyond the immediate recovery stage to address and support the long‐term complexities and impactof the condition. A significant proportion of participants (44.3%) identified unmet care needs, reinforcing the necessity for ongoing LC service provision that is underscored by the importance of holistic, patient‐centred approaches to care, acknowledging the non‐linear recovery and uncertain trajectory of this complex condition.

The persistent clinical and socio‐economic burden of LC is clear, with only 20.8% of participants working at pre‐infection capacity. Policymakers and service leaders should act to close the gap between LC service provision and patient need. There is a critical window of opportunity for ICBs to redesign services in collaboration with and informed by the patients' voice, learning from local contexts and ensuring care models are equitable, responsive and integrated. Without this, the burden of LC will continue to fall on individuals and families alone.

## Author Contributions

**Cassie Lee:** conceptualisation, investigation, writing – original draft, writing – review and editing, project administration, data curation, methodology, visualisation, formal analysis, validation, resources. **Paul Williams:** investigation, writing – original draft, writing – review and editing, methodology, formal analysis, data curation, visualisation, validation, project administration, resources. **Amiad Abrahams:** conceptualisation, investigation, methodology, writing – review and editing, writing – original draft, formal analysis, visualisation, validation, resources. **Julie Darbyshire:** conceptualisation, methodology, writing – review and editing, formal analysis, visualisation, validation, resources. **Helen E. Davies:** investigation, writing – review and editing, data curation, validation, visualisation, formal analysis. **Johannes De Kock:** writing – review and editing, writing – original draft, validation, visualisation. **Umut Esmer:** conceptualisation, writing – review and editing, methodology, formal analysis, visualisation, validation, resources. **Samantha A. Jones:** investigation, data curation, validation, visualisation. **Vicky Newey:** conceptualisation, visualisation, formal analysis, methodology, validation, resources. **Janet Scott:** writing – review and editing, formal analysis. **Nikki Smith:** writing – review and editing, validation, visualisation. **Darren Winch:** writing – review and editing, validation, visualisation. **Harsha Master:** validation, visualisation, writing – review and editing, investigation, supervision, writing – original draft. **Sarah Elkin:** conceptualisation, funding acquisition, supervision, formal analysis, methodology, validation, investigation, writing – review and editing, methodology, resources.

## LOCOMOTION Consortium Group

Nawar Bakerly, Principal Investigator; Kumaran Balasundaram, NHS Clinical Research Fellow; Megan Ball, NHS Clinical Research Fellow; Mauricio Barahona, Co‐Investigator; Alexander Casson, Co‐Investigator; Jonathan Clarke, HEI Researcher; Karen Cook, Patient Advisory Group Member; Rowena Cooper, NHS Clinical Research Fellow; Vasa Curcin, Co‐Investigator; Julie Darbyshire, Co‐Investigator; Helen Davies, Principal Investigator; Helen Dawes, Co‐Investigator; Simon de Lusignan, Co‐Investigator; Brendan Delaney, Chief Investigator; Carlos Echevarria, Principal Investigator; Sarah Elkin, Principal Investigator; Ana Belen Espinosa Gonzalez, HEI Researcher; Rachael Evans, Principal Investigator; Sophie Evans, Patient Advisory Group Member; Zacchaeus Falope, Principal Investigator; Ben Glampson, HEI Researcher; Madeline Goodwin, Research Assistant; Trish Greenhalgh, Co‐Investigator; Darren C. Greenwood, Co‐Investigator; Stephen Halpin, Principal Investigator; Juliet Harris, NHS Research Assistant; Will Hinton, HEI Researcher; Mike Horton, Co‐Investigator; Samantha Jones, NHS Clinical Research Fellow; Joseph Kwon, HEI Researcher; Cassie Lee, NHS Clinical Research Fellow; Ashliegh Lovett, NHS Clinical Research Fellow; Mae Mansoubi, HEI Researcher; Victoria Masey, NHS Clinical Research Fellow; Harsha Master, Principal Investigator; Erik Mayer, HEI Researcher; Bernardo Meza‐Torres, HEI Researcher; Ruairidh Milne, Patient Advisory Group Member; Ghazala Mir, Co‐Investigator; Jacqui Morris, Principal Investigator; Adam Mosley, NHS Research Assistant; Jordan Mullard, HEI Researcher; Daryl O'Connor, Co‐Investigator; Rory O'Connor, Co‐Investigator; Thomas Osborne, Project Manager; Amy Parkin, NHS Clinical Research Fellow; Stavros Petrou, Co‐Investigator; Anton Pick, Principal Investigator; Denys Prociuk, HEI Researcher; Clare Rayner, Patient Advisory Group Member; Amy Rebane, Patient and Public Involvement Manager; Natalie Rogers, Patient Advisory Group Member; Janet Scott, Principal Investigator; Manoj Sivan, Chief Investigator; Nikki Smith, Patient Advisory Group Member; Adam Smith, Statistician; Emma Tucker, Principal Investigator; Ian Tucker‐Bell, Patient Advisory Group Member; Paul Williams, NHS Clinical Research Fellow; Darren Winch, Patient Advisory Group Member; and Conor Wood, NHS Research Assistant.

## Disclosure

The views expressed in this publication are those of the author(s) and not necessarily those of NIHR or the Department of Health and Social Care.

## Ethics Statement

The LOCOMOTION study was approved by the Health Research Authority (HRA), which protects the interests of patients in health research, and the Yorkshire and Humber Leeds Bradford Research Ethics Committee (REC ref: 21/YH/0276).

## Consent

Explicit patient consent was not sought for the specific work; implied consent was assumed by completing the survey.

## Conflicts of Interest

All authors have completed the ICMJE uniform disclosure form at www.icmje.org/coi_disclosure.pdf and declare the following: C.L., P.L., J.S., and H.M. received financial support from the Locomotion Study. C.L. was granted travel expenses by the Locomotion study to present at an international conference. H.M. holds a Service Lead Position within the Covid Clinical Society with payment via host institution, has received honorarium for an article in Guidelines in Practice for Primary Care and has delivered various study days and presentations with either travel expenses paid and/or honorarium.

## Permission to Reproduce Material From Other Sources

The authors have nothing to report.

## Supporting information

Supplementary file 1_ Additional survey details.

Supplementary file 2 ‐ Online survey ‐ V3 ‐ deanonymised ‐ Clean.

Supplementary file 3 ‐ Qualitative Analysis ‐ V2 Clean.

Supplementary file 4_ Additional tables and charts ‐ V3 Clean.

## Data Availability

Data supporting this publication are owned by the individual sites, and the anonymised data by the University of Leeds, the project sponsor. The data are not publicly available due to privacy or ethical restrictions.

## References

[1] “COVID‐19 Rapid Guideline: Managing the Long‐Term Effects of COVID‐19 NICE Guideline,” accessed September 20, 2024, https://www.nice.org.uk/guidance/ng188. 33555768

[2] R. Chou , E. Herman , A. Ahmed , et al., “Long COVID Definitions and Models of Care,” Annals of Internal Medicine 177, no. 7 (July 2024): 929–940, 10.7326/M24-0677. 38768458

[3] R. De Luca , M. Bonanno , C. Rifici , A. Quartarone , and R. S. Calabrò , “Post‐Traumatic Olfactory Dysfunction: A Scoping Review of Assessment and Rehabilitation Approaches,” Frontiers in Neurology 14 (July 2023): 1193406, 10.3389/fneur.2023.1193406. 37521284 PMC10374209

[4] H. E. Davis , G. S. Assaf , L. McCorkell , et al., “Characterizing Long COVID in an International Cohort: 7 Months of Symptoms and Their Impact,” eClinicalMedicine 38 (2021): 101019, 10.1016/j.eclinm.2021.101019. 34308300 PMC8280690

[5] World Health Organization , “A Clinical Case Definition of Post COVID‐19 Condition by a Delphi Consensus, 6 October 2021,” accessed August 7, 2024, https://iris.who.int/handle/10665/345824.

[6] C. E. Hastie , D. J. Lowe , A. McAuley , et al., “True Prevalence of Long‐COVID in a Nationwide, Population Cohort Study,” Nature Communications 14, no. 1 (November 2023): 7892, 10.1038/s41467-023-43661-w. PMC10689486 38036541

[7] “Self‐Reported Coronavirus (COVID‐19) Infections and Associated Symptoms, England and Scotland: November 2023 to March 2024,” Office for National Statistics, accessed September 20, 2024, https://www.ons.gov.uk/peoplepopulationandcommunity/healthandsocialcare/conditionsanddiseases/articles/selfreportedcoronaviruscovid19infectionsandassociatedsymptomsenglandandscotland/november2023tomarch2024.

[8] “National Center for Health Statistics. U.S. Census Bureau, Household Pulse Survey, 2022–2024. Long COVID,” CDC, accessed August 12, 2024, https://www.cdc.gov/nchs/covid19/pulse/long-covid.htm.

[9] “The NHS Plan for Improving Long Covid Services,” NHS England, accessed August 7, 2024, https://www.england.nhs.uk/wp-content/uploads/2022/07/C1607_The-NHS-plan-for-improving-long-COVID-services_July-2022.pdf.

[10] “NHS Sets Out Long COVID Action Plan for Thousands of People With Persistent Symptom,” NHS England, accessed September 20, 2024, https://www.england.nhs.uk/2022/07/nhs-sets-out-long-covid-action-plan-for-thousands-of-people-with-persistent-symptom/.

[11] E. Duncan , L. Alexander , J. Cowie , et al., “Investigating Scottish Long COVID Community Rehabilitation Service Models From the Perspectives of People Living With Long COVID and Healthcare Professionals: A Qualitative Descriptive Study,” BMJ Open 13, no. 12 (2023): e078740, 10.1136/bmjopen-2023-078740. PMC10729197 38101833

[12] T. Greenhalgh , J. L. Darbyshire , C. Lee , E. Ladds , and J. Ceolta‐Smith , “What Is Quality in Long Covid Care? Lessons From a National Quality Improvement Collaborative and Multi‐Site Ethnography,” BMC Medicine 22, no. 1 (April 2024): 1–21, 10.1186/S12916-024-03371-6. 38616276 PMC11017565

[13] E. Ladds , A. Rushforth , S. Wieringa , et al., “Developing Services for Long COVID: Lessons From a Study of Wounded Healers,” Clinical Medicine 21, no. 1 (January 2021): 59–65, 10.7861/clinmed.2020-0962. 33479069 PMC7850205

[14] H. Lewthwaite , A. Byrne , B. Brew , and P. G. Gibson , “Treatable Traits for Long COVID,” Respirology 28, no. 11 (November 2023): 1005–1022, 10.1111/resp.14596. 37715729

[15] S. Rajan , K. Khunti , N. Alwan , et al., “In the Wake of the Pandemic: Preparing for Long COVID Health Systems and Policy Analysis,” 2021, accessed September 20, 2024, https://www.ncbi.nlm.nih.gov/books/NBK569598/pdf/Bookshelf_NBK569598.pdf. 33877759

[16] C. M. Van Der Feltz‐Cornelis , J. Sweetman , F. Turk , et al., “Integrated Care Policy Recommendations for Complex Multisystem Long Term Conditions and Long COVID,” Antony Loveless 10: 16, 10.1038/s41598-024-64060-1. PMC11176166 38871773

[17] “Integrated Care Systems: Design Framework 2021,” NHS England and NHS Improvement 2021, accessed September 20, 2024, https://www.england.nhs.uk/wp-content/uploads/2021/06/B0642-ics-design-framework-june-2021.pdf.

[18] “Commissioning Guidance for Post COVID Services for Adults, Children, and Young People,” NHS England, accessed August 22, 2024, https://www.england.nhs.uk/wp-content/uploads/2022/07/PRN00488i-commissioning-guidance-for-post-covid-services-for-adults-children-and-young-people-v4.pdf.

[19] F. Turk , J. Sweetman , C. CHew‐Graham , et al., “Accessing Care for Long Covid From the Perspectives of Patients and Healthcare Practitioners: A Qualitative Study,” (2024), 10.1111/hex.14008. PMC10938067 38481384

[20] D. Sunkersing , M. Ramasawmy , N. A. Alwan , et al., “What Is Current Care for People With Long COVID in England? A Qualitative Interview Study,” BMJ Open 14, no. 5 (2024): e080967, 10.1136/bmjopen-2023-080967. PMC11107429 38760030

[21] C. Fang , S. A. Baz , L. Sheard , and J. D. Carpentieri , “‘They Seemed to Be Like Cogs Working in Different Directions’: A Longitudinal Qualitative Study on Long COVID Healthcare Services in the United Kingdom From a Person‐Centred Lens,” BMC Health Services Research 24, no. 1 (April 2024): 406, 10.1186/s12913-024-10891-7. 38561719 PMC10986002

[22] T. Kingston , A. Taylor , A. O'Donnell , H. Atherton , D. Blane , and C. Chew‐Graham , “Finding the ‘Right’ GP: A Qualitative Study of the Experiences of People With Long‐COVID,” (December 2020), 10.3399/bjgpopen20X101143. PMC7880173 33051223

[23] F. J. Leggat , C. Heaton‐Shrestha , J. Fish , et al., “An Exploration of the Experiences and Self‐Generated Strategies Used When Navigating Everyday Life With Long Covid,” BMC Public Health 24, no. 1 (March 2024): 789, 10.1186/s12889-024-18267-6. 38481230 PMC10938753

[24] Healthwatch England , “What People Have Told Us About Long Covid,” 2020, accessed September 20, 2024, https://www.healthwatch.co.uk/sites/healthwatch.co.uk/files/20220225_Long%20Covid%20Evidence%20Reviewv.2_0.pdf.

[25] L. D. Hawke , A. Nguyen , N. Y. Sheikhan , et al., “Swept Under the Carpet: A Qualitative Study of Patient Perspectives on Long COVID, Treatments, Services, and Mental Health,” BMC Health Services Research 23, no. 1 (October 2023): 1088, 10.1186/s12913-023-10091-9. 37821939 PMC10568931

[26] M. Sivan , D. Greenwood , A. Smith , R. R. L. Roman , T. Osborne , and M. Goodwin , “A National Evaluation of Outcomes in Long COVID Services Using Digital PROM Data From the ELAROS Platform,” (October 2023), accessed September 20, 2024, https://locomotion.leeds.ac.uk/wp-content/uploads/sites/74/2023/10/National-Evaluation-of-LC-Service-Outcomes-using-ELAROS-Data-09-10-23.pdf.

[27] E. Duan , K. Garry , L. I. Horwitz , and H. Weerahandi , “‘I Am Not the Same as I Was Before’: A Qualitative Analysis of COVID‐19 Survivors,” International Journal of Behavioural Medicine 30, no. 5 (October 2023): 663–672, 10.1007/s12529-022-10129-y. PMC9559269 36227557

[28] G. Schaap , M. Wensink , C. J. M. Doggen , J. van der Palen , H. E. Vonkeman , and C. Bode , “‘It Really Is an Elusive Illness’—Post‐COVID‐19 Illness Perceptions and Recovery Strategies: A Thematic Analysis,” International Journal of Environmental Research and Public Health 19, no. 20 (2022): 13003, 10.3390/ijerph192013003. 36293582 PMC9602798

[29] C. E. Kennelly , A. Nguyen , N. Y. Sheikhan , et al., “The Lived Experience of Long COVID: A Qualitative Study of Mental Health, Quality of Life, and Coping,” PLoS One 18, no. 10 (2023): e0292630, 10.1371/JOURNAL.PONE.0292630. 37831706 PMC10575511

[30] M. Sivan , T. Greenhalgh , J. L. Darbyshire , et al., “LOng COvid Multidisciplinary Consortium Optimising Treatments and Services AcrOss the NHS (LOCOMOTION): Protocol for a Mixed‐Methods Study in the UK,” BMJ Open 12, no. 5 (2022): e063505, 10.1136/bmjopen-2022-063505. PMC9114312 35580970

[31] J. Darbyshire , T. Greenhalgh , N. D. Bakerly , et al., “Improving Quality in Adult Long Covid Services: Findings From the LOCOMOTION Quality Improvement Collaborative,” Clinical Medicine 24, no. 5 (2024): 100237, 10.1016/j.clinme.2024.100237. 39181334 PMC11421994

[32] S. J. Kamper , C. G. Maher , and G. Mackay , “Global Rating of Change Scales: A Review of Strengths and Weaknesses and Considerations for Design,” Journal of Manual & Manipulative Therapy 17, no. 3 (July 2009): 163–170, 10.1179/jmt.2009.17.3.163. 20046623 PMC2762832

[33] F. V. C. Machado , R. Meys , J. M. Delbressine , et al., “Construct Validity of the Post‐COVID‐19 Functional Status Scale in Adult Subjects With COVID‐19,” Health and Quality of Life Outcomes 19, no. 1 (December 2021): 40, 10.1186/s12955-021-01691-2. 33536042 PMC7856622

[34] V. Braun and V. Clarke , “Using Thematic Analysis in Psychology,” Qualitative Research in Psychology 3, no. 2 (January 2006): 77–101, 10.1191/1478088706qp063oa.

[35] O. C. Robinson , “Conducting Thematic Analysis on Brief Texts: The Structured Tabular Approach,” Qualitative Psychology 9, no. 2 (June 2022): 194–208, 10.1037/qup0000189.

[36] J. Darbyshire , “Sleep in the Intensive Care Unit: Limiting Elements of Noise in the Critical Care Environment (SILENCE).” (PhD diss., University of Oxford, 2019).

[37] J. W. Creswell and V. L. P. Clark , Designing and Conducting Mixed Methods Research, 2nd ed. (SAGE Publications, Inc., 2011).

[38] K. E. J. Philip , H. Owles , S. McVey , et al., “An Online Breathing and Wellbeing Programme (ENO Breathe) for People With Persistent Symptoms Following COVID‐19: A Parallel‐Group, Single‐Blind, Randomised Controlled Trial,” Lancet Respiratory Medicine 10, no. 9 (September 2022): 851–862, 10.1016/S2213-2600(22)00125-4. 35489367 PMC9045747

[39] M. Sivan , A. B. Smith , T. Osborne , et al., “Long Covid Clinical Severity Types Based on Symptoms and Functional Disability: A Longitudinal Evaluation,” 10.2139/SSRN.4642650. PMC11012375 38610673

[40] J. B. Soriano , S. Murthy , J. C. Marshall , P. Relan , and J. V. Diaz , A Clinical Case Definition of Post‐COVID‐19 Condition by a Delphi Consensus (Elsevier Ltd., 2022), 10.1016/S1473-3099(21)00703-9. PMC8691845 34951953

[41] R. J. O'Connor , A. Parkin , G. Mir , et al., “Work and Vocational Rehabilitation for People Living With Long Covid,” BMJ 385 (2024): e076508, 10.1136/bmj-2023-076508. 38729647

[42] J. Kwon , R. Milne , C. Rayner , et al., “Impact of Long COVID on Productivity and Informal Caregiving,” European Journal of Health Economics 1: 3, 10.1007/s10198-023-01653-z. PMC11377524 38146040

[43] S. A. Baz , C. Fang , J. D. Carpentieri , and L. Sheard , “‘I Don't Know What to Do or Where to Go’. Experiences of Accessing Healthcare Support From the Perspectives of People Living With Long Covid and Healthcare Professionals: A Qualitative Study in Bradford, UK,” Health Expectations 26, no. 1 (February 2023): 542–554, 10.1111/hex.13687. 36512382 PMC10124541

[44] K. Macpherson , K. Cooper , J. Harbour , D. Mahal , C. Miller , and M. Nairn , “Experiences of Living With Long COVID and of Accessing Healthcare Services: A Qualitative Systematic Review,” BMJ Open 12, no. 1 (2022): e050979, 10.1136/bmjopen-2021-050979. PMC8753091 35017239

[45] I. Schaurer and B. Weiß , “Investigating Selection Bias of Online Surveys on Coronavirus‐Related Behavioral Outcomes,” Survey Research Methods 14, no. 2 (2020): 103–108, 10.18148/srm/2020.v14i2.7751.

