# Supplementary material for: What Can We Learn Four Years On? A Multi‐Centre Service Evaluation Exploring Symptoms, Functional Impact, Recovery and Care Pathways in Long Covid
Source: Health Expect. 2025 Nov 6;28(6):e70435. doi: 10.1111/hex.70435 (PMC12592685; doi:10.1111/hex.70435)
Supplement: Supplementary file 1 — _ Additional survey details. [file HEX-28-e70435-s002.docx]

### Supplementary file 1 – Additional survey details

### Text message to participants

Dear [PATEINT NAME],

Do you still experience Long Covid symptoms, are you better or not?  We are reviewing our service and would like to hear how you are now. [LINK TO FORM HERE]

Your unique ID number is: [INSERT PATIENT UNIQUE ID NUMBER HERE]

 Please completed this short survey within the next 7 days if you are happy to share your experiences.

Thank you

 The [SITE NAME] Post Covid Assessment Service

### Summary description of survey questions

- **Questions 2, 3, 4**, **LC functional impact and symptoms:** Including the Post Covid Functional scale (PCFS) [34]. The scale includes a Likert scale from 0 - 4, where a score of 0 relates to experiencing no symptoms related to COVID-19 infection.
  - A score of 1 relates to Negligible limitations i.e. the individual feels they can perform all usual duties/activities; however, they experience persistent symptoms e.g. cough, loss of taste/smell.
  - A score of 2 relates to slight limitations i.e. occasionally needing to avoid or reduce usual duties e.g. activities or work and may need occasional assistance to complete activities due to persistent symptoms.
  - A score of 3 relates to moderate limitations i.e. being unable to perform all usual duties or activities including work due to symptoms.
  - A score of 4 relates to severe limitations i.e. being unable to take care of oneself and therefore, being dependent on nursing care or assistance from another person due to symptoms.
- **Questions 5, 6, 7,** **recovery and trajectory (prior six months):** A 3-point global rate of change scale [34] and two follow-up questions exploring what aided recovery
- **Questions 8, 9, 10, 11, 12,** **LC local service specific:** Predominantly free text open questions suggesting areas of improvement with identification of local service onward referrals actions.
- **Questions 13, 14, 15, 16**, **LC healthcare**. Current care/support status and perceived unmet healthcare needs, included free text follow-up questions
- **Question 17: Vocational status**
- **Question 18: Follow-up free text comments**

### Table of survey parameters

| Q No | Topic | Question | Parameters |
| --- | --- | --- | --- |
|  |  |  |  |
| 2 | Impact of LC - Function | How much are you currently impacted by Long Covid in your everyday life? | Post-Covid Function Scale, rating 0-4. |
| 3* | Impact of LC - Today | Is today a good or bad day for you? | Optional question for those functional limitations.  VAS star rating 1-5, where 5 is “good-day” |
| 4 | Impact of LC - Symptoms | What are the main on-going Long Covid symptoms you are still experiencing? | Optional question for those functional limitations.  Max 5 options from list of 16, plus “other” option  (this was increased from 3 during the pilot phase) |
| 5 | Recovery | Process of recovery | Optional question for those functional limitations  3 options |
| 6 | Recovery | What do you think has helped you recover? | Optional question for those some recovery  3 options pus “other” – all that apply |
| 7 | Recovery | What has helped you most | Optional question for those some recovery  Follow-up free text question |
| 8 | Local service specific | Rate the onward referrals, investigation, and plan. | Rating 1-5 stars and with |
| 9 | Local service specific | Why did you give it that rating? | Follow-up free text question if applicable |
| 10, | Local service specific | Were all the referrals completed | 4 options plus “other” |
| 11 | Local service specific |  | Optional question if not all actions completed. Follow-up free text question |
| 12 | Local service specific Expectations | Is there anything else you think we should have done for you? | Free text question |
| 13 | Care utilisation | Are you currently receiving care/support | 3 options plus “other” with follow-up question of |
| 14 | Care utilisation | What support/care? | Optional question if receiving care/support  8 options plus “other”– multi options |
| 15 | On-going care needs | Do you feel you need any additional support/care for LC? | 2 options plus “N/A recovered” |
| 16 | On-going care needs | What additional support/care? | Optional question feel need additional support Follow-up free text question |
| 17 | Impact of LC - Vocation | Your current working / vocational situation | 8 options plus “other” free text |
| 18 | Follow-up | Any further comments | Unlimited free text |
| 19, 20 | Survey method | How have you completed this form? | 2 options, and follow-up question on actions from telephone call option only. |

- Question was suggested by the PPI members during design phase, to acknowledge the fluctuating nature of LC
