# Supplementary figures and images for: What Can We Learn Four Years On? A Multi‐Centre Service Evaluation Exploring Symptoms, Functional Impact, Recovery and Care Pathways in Long Covid
Source: Health Expect. 2025 Nov 6;28(6):e70435. doi: 10.1111/hex.70435 (PMC12592685; doi:10.1111/hex.70435)

### Supplementary file 2 - On-line survey


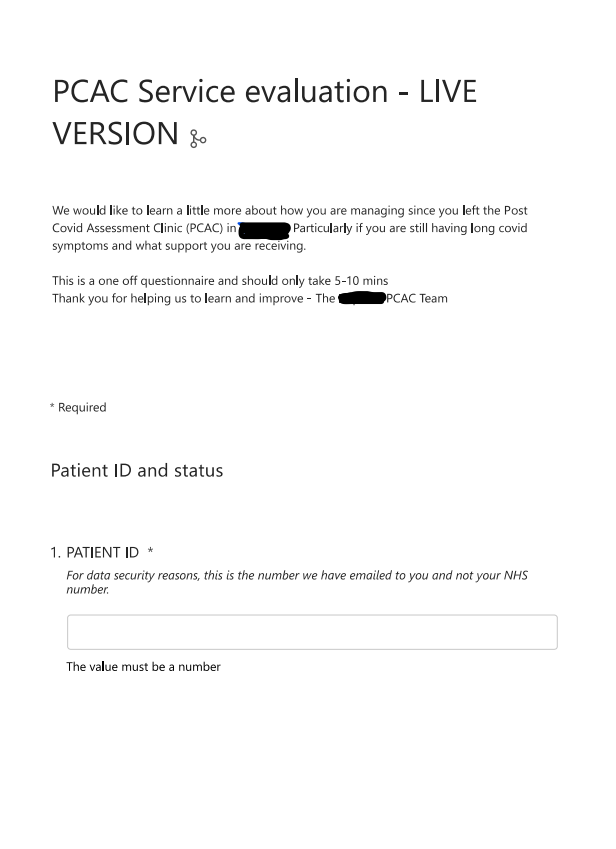


[service] name]

[service].


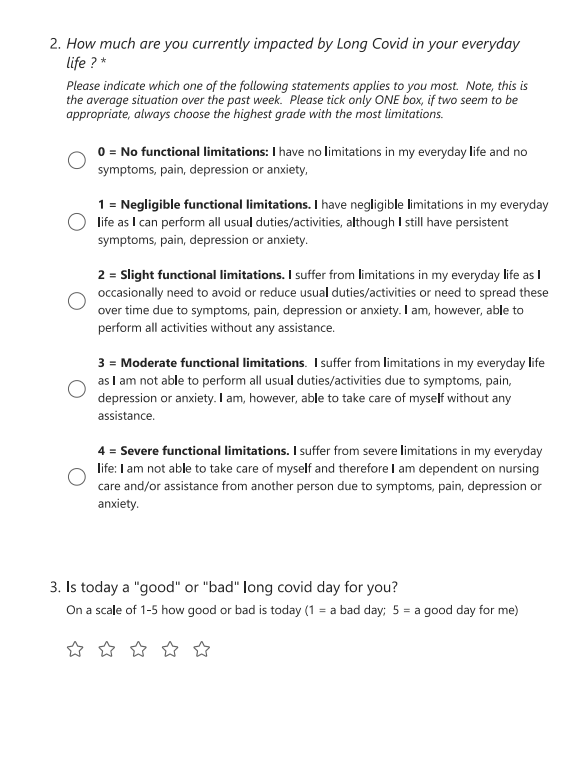


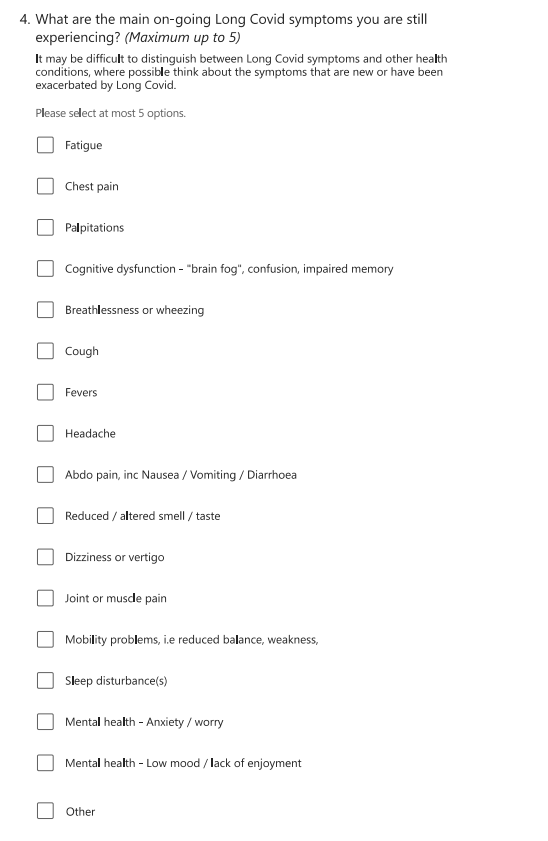


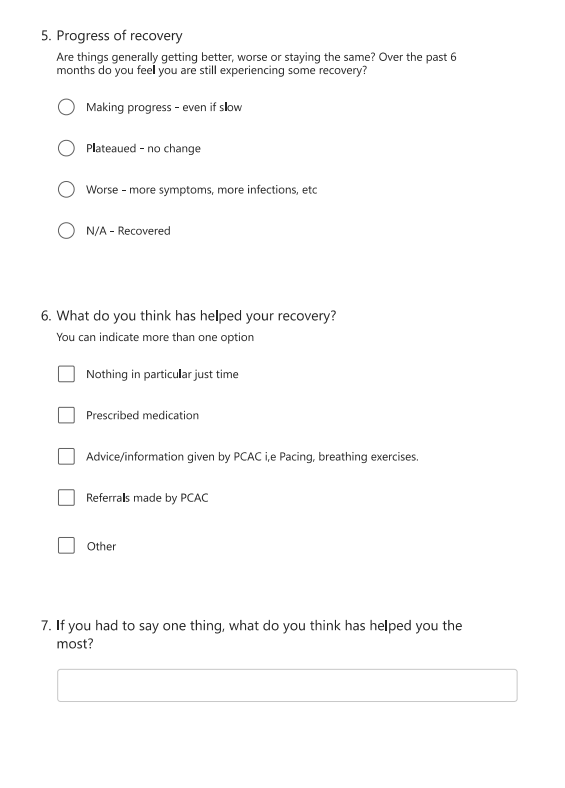


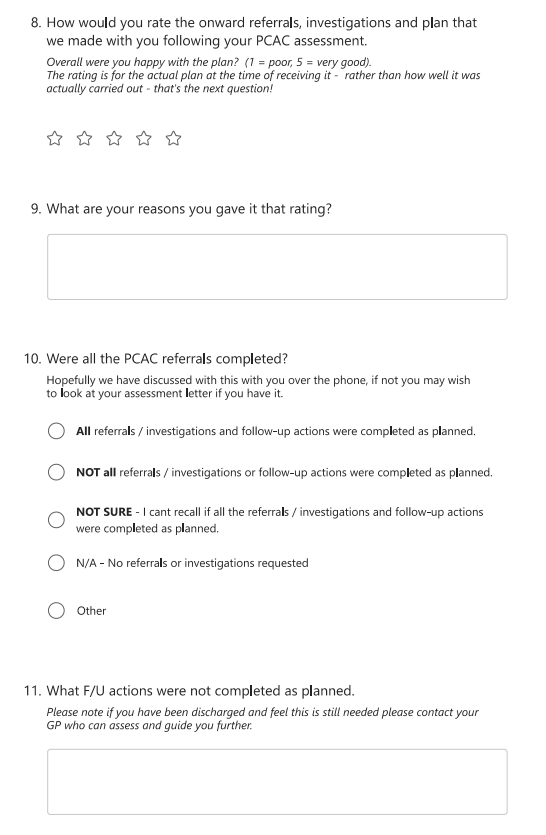


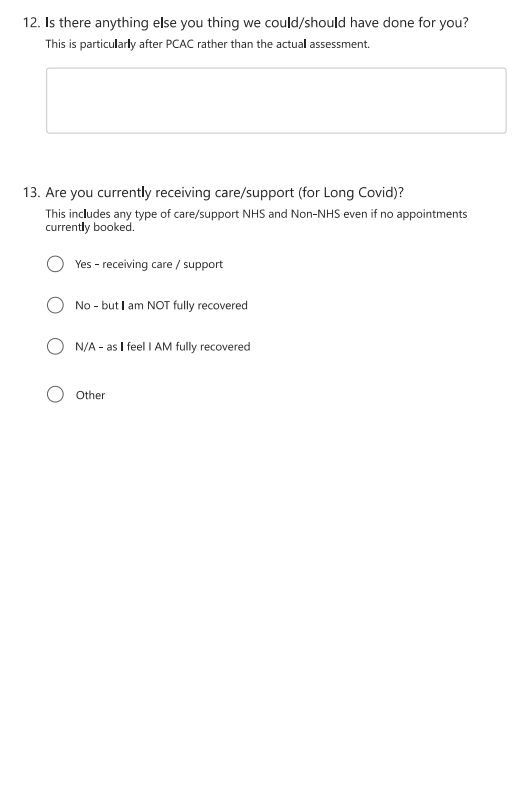


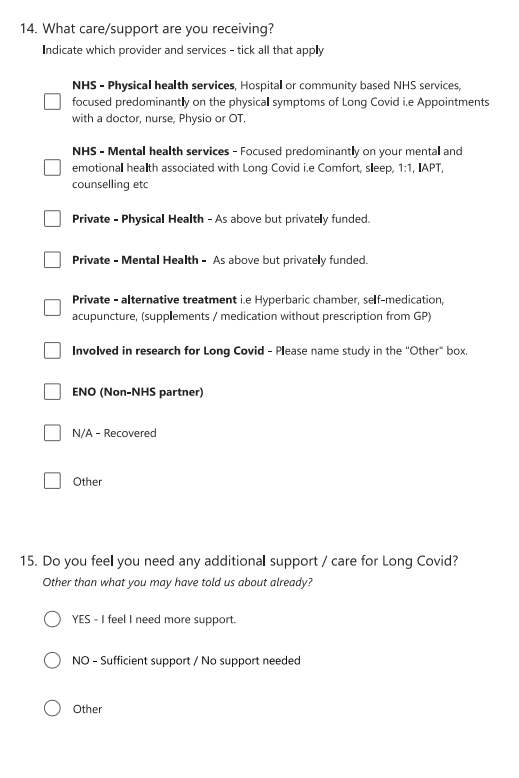


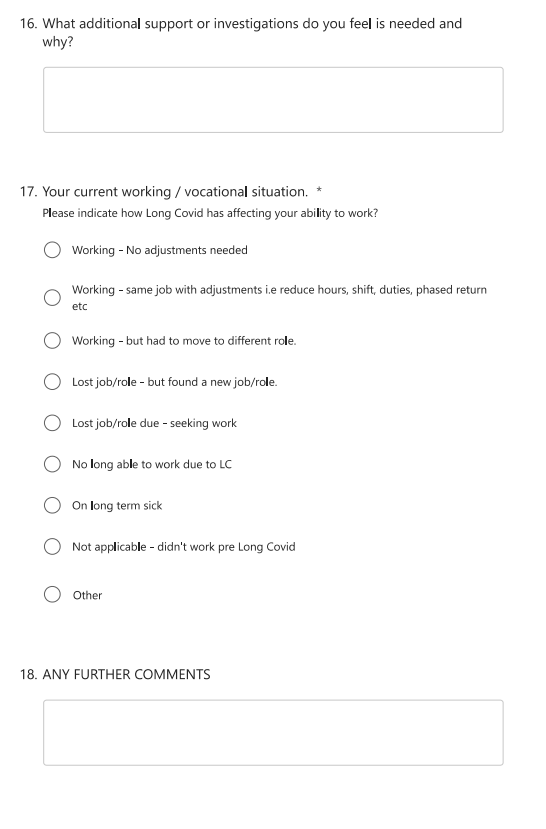


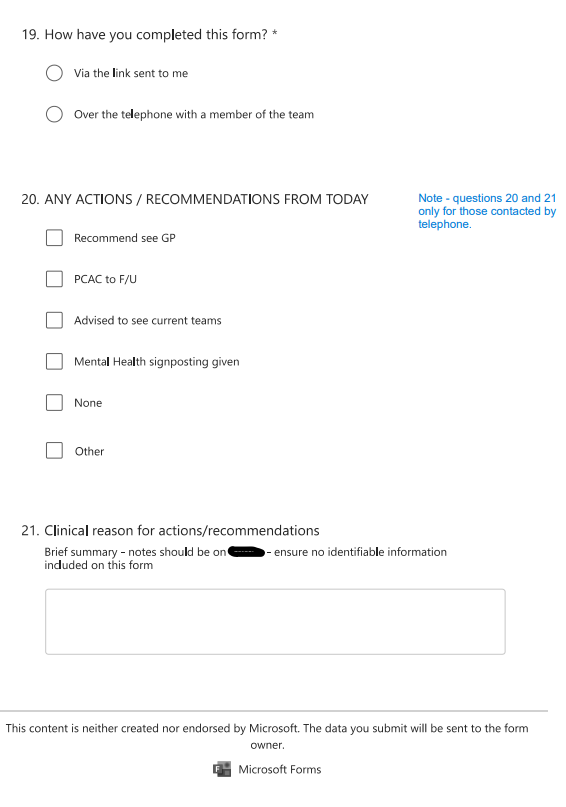


[system]

Supplement: Supplementary file 2 — ‐ Online survey ‐ V3 ‐ deanonymised ‐ Clean. [file HEX-28-e70435-s004.docx]
