# Supplementary material for: What Can We Learn Four Years On? A Multi‐Centre Service Evaluation Exploring Symptoms, Functional Impact, Recovery and Care Pathways in Long Covid
Source: Health Expect. 2025 Nov 6;28(6):e70435. doi: 10.1111/hex.70435 (PMC12592685; doi:10.1111/hex.70435)
Supplement: Supplementary file 3 — ‐ Qualitative Analysis ‐ V2 Clean. [file HEX-28-e70435-s003.docx]

### Supplementary file 3 - Qualitative Analysis

Coding

Refers to the processes of breaking down the data into discrete parts, linking data, categorising the data, establishing categories and subcategories, making links between the data categories and of identifying one or more overarching categories.

Step 1: “Open” coding

The process of breaking down the data into discrete parts (individual data items), by word, by phrase, by sentence, by paragraph – by listening to and reading the transcript of each interview, extracting from the data set the words and phrases used by the interviewees, and by repeating this process until no further discrete data items emerge (are identified) as a result of further reading – this point is referred to as ‘saturation’.

Step 2: “Axial” coding

The process of linking data, categorising the data, establishing categories and subcategories and making links between the data categories. Clustering of data in this way involves a number of processes:

• examining individual data items

• establishing and defining categories and subcategories

• locating individual data items within categories and subcategories

• constantly comparing data items both within individual data sets (single interviews) and between data sets including comparisons within and between categories and subcategories.

Step 3: “Selective” coding

The process of identifying one or more overarching categories, establishing the links between categories and subcategories both within and between the overarching categories – theory building – establishing the relationships between the data as a meaningful whole.
