# Supplementary material for: What Can We Learn Four Years On? A Multi‐Centre Service Evaluation Exploring Symptoms, Functional Impact, Recovery and Care Pathways in Long Covid
Source: Health Expect. 2025 Nov 6;28(6):e70435. doi: 10.1111/hex.70435 (PMC12592685; doi:10.1111/hex.70435)
Supplement: Supplementary file 4 — _ Additional tables and charts ‐ V3 Clean. [file HEX-28-e70435-s001.docx]

### Supplementary file 4 – Additional tables and charts

Table A1 - PCFS by patient characteristics, Infection data, LC clinic access, recovery and care provision

|  | **Responder** | | | | | | **Non-responder** | **All** |
| --- | --- | --- | --- | --- | --- | --- | --- | --- |
|  | **ALL Responders** | PCFS 0 | PCFS 1 | PCFS 2 | PCFS 3 | PCFS 4 |  |  |
|  | **269** | 16 | 27 | 76 | 113 | 37 | **244** | **513** |
| **Patient characteristics** |  |  |  |  |  |  |  |  |
| *Mean age (missing n=3)* | **52.67** | 50.8 | 53.6 | 53.1 | 51.8 | 54.7 | **49.41** | **51.1** |
| StdDev of Age | **12.02** | 13.6 | 13.2 | 12.2 | 10.7 | 13.2 | **13.60** | **12.9** |
| *Sex (female)* | **186** | 11 | 17 | 51 | 83 | 24 | **178** | **364** |
| *Sex (male)* | **80** | 5 | 10 | 25 | 27 | 13 | **66** | **146** |
| *Missing* | **3** | 0 | 0 | 0 | 3 | 0 | **0** | **3** |
| **Ethnicity** | | | | | | | | |
| White - British | 148 | 6 | 17 | 47 | 67 | 11 | 128 | 276 |
| White - Any other White background | 34 | 6 | 2 | 9 | 11 | 6 | 29 | 63 |
| Asian- Asian British  (inc British Indian/Pakistani) | 19 | 0 | 5 | 7 | 6 | 1 | 18 | 37 |
| Asian - Any Other Asian Background | 3 | 0 | 0 | 1 | 1 | 1 | 6 | 9 |
| Black or Black British - African/Caribbean | 9 | 2 | 0 | 2 | 3 | 2 | 12 | 21 |
| Mixed - Any other mixed background | 4 | 0 | 3 | 0 | 0 | 1 | 4 | 8 |
| Other Ethnic Groups - Any other ethnic group | 37 | 1 | 0 | 9 | 15 | 12 | 34 | 71 |
| Missing | 15 | 1 |  | 1 | 10 | 3 | 13 | 28 |
| Total | 269 | 6 | 17 | 47 | 67 | 11 | 244 | 513 |
| **Index of multiple deprivation (quintiles)** | | | | | | | | |
| *1* | 24 | 0 | 3 | 2 | 11 | 8 | 27 |  |
| *2* | 42 | 3 | 2 | 11 | 20 | 6 | 34 |  |
| *3* | 48 | 3 | 5 | 12 | 22 | 6 | 63 |  |
| *4* | 54 | 3 | 6 | 20 | 21 | 4 | 60 |  |
| *5* | 79 | 6 | 11 | 25 | 28 | 9 | 47 |  |
| *Missing* | 22 | 1 | 0 | 6 | 11 | 4 | 13 |  |
| *Total* | 269 | 16 | 27 | 76 | 113 | 37 | 244 |  |
| **Predominant variance at time of infection** | | | | | | | | |
| Pre-Alpha | **113** | 9 | 5 | 39 | 43 | 17 | **111** | **224** |
| Alpha | **66** | 4 | 8 | 13 | 34 | 7 | **46** | **112** |
| Delta | **25** | 0 | 4 | 6 | 11 | 4 | **21** | **46** |
| Omicron | **25** | 1 | 3 | 9 | 10 | 2 | **10** | **35** |
| Missing | **40** | 2 | 7 | 9 | 15 | 7 | **56** | **96** |
| Total | **269** | *16* | *27* | *76* | *113* | *37* | **244** | **513** |
| ***Mean in duration in days of LC*** | **1204.40** | 1261.79 | 1103.55 | 1229.88 | 1195.30 | 1217.10 | **1262.15** | **1230.54** |
| *SD* | **275.74** | 245.57 | 280.70 | 281.19 | 280.49 | 255.39 | **252.62** | **266.77** |
| **Severity of infection (n=306 as data for two sites only known, the third site predominantly community referrals)** | | | | | | |  |  |
| Community | **144** | 12 | 9 | 39 | 65 | 19 | **76** | **220** |
| Hospital | **18** | 0 | 2 | 4 | 6 | 6 | **10** | **28** |
| Hospital-ITU | **4** | 0 | 0 | 0 | 1 | 3 | **2** | **6** |
| Missing | **4** | 1 | 0 | 0 | 3 | 0 | **46** | **50** |
| Vaccine triggered | **1** | 0 | 0 | 0 | 1 | 0 | **1** | **2** |
| **Grand Total** | **171** | **13** | **11** | **43** | **76** | **28** | **135** | **306** |
| **Duration since first assessment LC clinic** | | | | | | | | |
| less than 6 months | **30** | 1 | 3 | 11 | 13 | 2 | **22** | **52** |
| 6-12 months | **76** | 1 | 11 | 21 | 37 | 6 | **86** | **162** |
| 12-18 months | **49** | 4 | 3 | 17 | 18 | 7 | **27** | **76** |
| 18-24 months | **37** | 4 | 1 | 8 | 19 | 5 | **27** | **64** |
| 24-30 months | **20** | 3 | 1 | 5 | 6 | 5 | **17** | **37** |
| 30-36 months | **18** | 1 | 1 | 6 | 5 | 5 | **5** | **23** |
| more than 3y | **1** | 0 | 0 | 0 | 1 | 0 | **7** | **8** |
| Missing | **38** | 2 | 7 | 8 | 14 | 7 | **53** | **91** |
| Total | **269** | **16** | **27** | **76** | **113** | **37** | **244** | **513** |
| **Site** |  |  |  |  |  |  |  |  |
| *Site A* | **99** | 9 | 4 | 24 | 40 | 22 | **90** | **189** |
| *Site B* | **72** | 4 | 7 | 19 | 36 | 6 | **45** | **117** |
| *Site C* | **98** | 3 | 16 | 33 | 37 | 9 | **109** | **207** |
| Total | **269** | **16** | **27** | **76** | **113** | **37** | **244** | **513** |
| **Progress of recovery (251 out of 269 answered)** | |  |  |  |  |  |  |  |
| N/A - Recovered | **5** | 0 | 3 | 1 | 1 |  | **N/A** | **N/A** |
| Making progress - even if slow | **96** | 0 | 15 | 40 | 33 | 8 | **N/A** | **N/A** |
| Plateaued - no change | **100** | 0 | 9 | 33 | 48 | 10 | **N/A** | **N/A** |
| Worse - more symptoms, more infections | **50** | 0 | 0 | 2 | 29 | 19 | **N/A** | **N/A** |
| (blank) | **18** | **16** |  |  | **2** |  | **N/A** | **N/A** |
| **What aided recovery** | | | | | | | | |
| Nothing in particular just time | **50** | 7 | 7 | 19 | 15 | 2 | **N/A** | **N/A** |
| Prescribed medication | **25** | 2 | 6 | 9 | 5 | 3 | **N/A** | **N/A** |
| Advice/information given by LC clinic | **46** | 4 | 8 | 21 | 10 | 3 | **N/A** | **N/A** |
| Referrals made by LC clinic | **18** | 1 | 3 | 7 | 6 | 1 | **N/A** | **N/A** |
| Total | **139** | **14** | **24** | **56** | **36** | **9** | **N/A** | **N/A** |
| **Current care provision** | | | | | | | | |
| N/A - as I feel I AM fully recovered (n=25) | **25** | 13 | 8 | 3 | 1 |  | **N/A** | **N/A** |
| Not receiving care /support - and NOT fully recovered (n=140) | **140** | 2 | 7 | 49 | 62 | 20 | **N/A** | **N/A** |
| Yes receiving care / support and NOT fully recovered (n=81) | **81** | 1 | 7 | 17 | 43 | 13 | **N/A** | **N/A** |
| None - Responder (n=244) | **N/A** | N/A | N/A | N/A | N/A | N/A | **N/A** | **N/A** |
| Grand Total (490) | **246** | 16 | 22 | 69 | 106 | 33 | **N/A** | **N/A** |

Table A2 - Comparison of characteristics and survey responses for vocational status and GroC, between those who do, and do not need additional care/support.

|  | **No, support**  (Sufficient support or no support needed) | | **Yes, need support**  (I feel I need more support) | |
| --- | --- | --- | --- | --- |
|  | N=75 | % | N=126 | % |
| Mean age | 54.6 | N/A | 50.5 | N/A |
| Age (SD) | 11.6 | N/A | 11.6 | N/A |
| * *Sex (female)* | 50 | 66.7% | 82 | 65.1% |
| * *Sex (male)* | 25 | 33.3% | 41 | 32.5% |
| Missing | 0 | 0% | 3 | 2.4% |
| ***Ethnicity** | | | | |
| White - British | 42 | 56.0% | 66 | 52.4% |
| White - Any other White background | 7 | 9.3% | 20 | 15.9% |
| Asian- Asian British  (inc British Indian/Pakistani) | 1 | 1.3% | 2 | 1.6% |
| Asian - Any Other Asian Background | 9 | 12.0% | 6 | 4.8% |
| Black or Black British - African/Caribbean | 2 | 2.7% | 5 | 4.0% |
| Mixed - Any other mixed background | 2 | 2.7% | 0 | 0.0% |
| Other Ethnic Groups - Any other ethnic group | 10 | 13.3% | 19 | 15.1% |
| Missing | 2 | 2.7% | 8 | 6.3% |
| Total | 75 | 100.0% | 126 | 100.0% |
| ***Index of multiple deprivation (quintiles)** | | | | |
| 1 | 7 | 9.3% | 11 | 8.7% |
| 2 | 9 | 12.0% | 21 | 16.7% |
| 3 | 13 | 17.3% | 22 | 17.5% |
| 4 | 14 | 18.7% | 25 | 19.8% |
| 5 | 30 | 40.0% | 31 | 24.6% |
| Missing | 2 | 2.7% | 16 | 12.7% |
| Total | 75 | 100.0% | 126 | 100.0% |
| ***Predominant UK variance at time of infection** | | | | |
| Pre-Alpha | 31 | 41.3% | 57 | 45.2% |
| Alpha | 14 | 18.7% | 31 | 24.6% |
| Delta | 7 | 9.3% | 11 | 8.7% |
| Omicron | 9 | 12.0% | 11 | 8.7% |
| Missing | 14 | 18.7% | 16 | 12.7% |
| **Total** | 75 | 100.0% | 126 | 100.0% |
| ***Severity of initial infection only provided by two sites.** | | | | |
| Community | 27 | 71.1% | 80 | 87.9% |
| Hospital-Ward | 7 | 18.4% | 7 | 7.7% |
| Hospital-ITU | 2 | 5.3% | 1 | 1.1% |
| Vaccine triggered | 1 | 2.6% | 0 | 0.0% |
| Missing | 1 | 2.6% | 3 | 3.3% |
| Total *(for two sites where data available)* | 38 | 100.0% | 91 | 100.0% |
| Sites did not provided data | 37 | N/A | 35 | N/A |
| Total | 75 |  | 126 |  |
| ***Duration of Long COVID** | | | | |
| <1 year | 0 | 0.0% | 0 | 0.0% |
| 1-2 years | 3 | 4.0% | 9 | 7.1% |
| 2-3 years | 19 | 25.3% | 19 | 15.1% |
| 3-4 years | 23 | 30.7% | 55 | 43.7% |
| >4 years | 16 | 21.3% | 29 | 23.0% |
| Missing | 14 | 18.7% | 14 | 11.1% |
| **Total** | 75 | 100.0% | 126 | 100.0% |
| Average of Duration of LC | 1196 | N/A | 1223 | N/A |
| StdDev of Duration of LC | 294 | N/A | 263 | N/A |
| ***Interval since first assessment at LC clinic** | | | | |
| <1 year | 28 | 37.3% | 46 | 36.5% |
| 1-2 years | 21 | 28.0% | 49 | 38.9% |
| 2-3 years | 8 | 10.7% | 16 | 12.7% |
| >3 years | 4 | 5.3% | 1 | 0.8% |
| Missing | 14 | 18.7% | 14 | 11.1% |
| **Total** | 75 | 100.0% | 126 | 100.0% |
| ****Vocational status** (n=151 who completed both questions) | | | | |
| Working - No adjustments needed | 16 | 53.3% | 14 | 46.7% |
| Working - with adjustments i.e reduce hours, shift, duties, phased return etc | 26 | 38.2% | 42 | 61.8% |
| No longer able to work due to LC | 10 | 27.8% | 26 | 72.2% |
| Not applicable - didn't work pre-Long COVID | 8 | 47.1% | 9 | 52.9% |
| Total6039.7%9160.3%Total | 60 | 39.7% | 91 | 60.3% |
| ****Progress (GroC)** (n=197 who completed both questions) | | | | |
| N/A - Recovered | 1 | 50.0% | 1 | 50.0% |
| Making progress - even if slow | 42 | 55.3% | 34 | 44.7% |
| Plateaued - no change | 19 | 25.0% | 57 | 75.0% |
| Worse - more symptoms, more infections, etc | 9 | 20.9% | 34 | 79.1% |
| Total | 71 | 36.0% | 126 | 64.0% |

*For characteristics and demographics percentage is calculated within group.

**For survey results, percentage is distribution across groups.

Figure A1 – PCFS distribution according to year of initial LC assessment date

Figure A2 - PCFS scale and Characteristic, infection type, mean duration of LC.

Figure A3 – Self-reported healthcare utilisation according to duration since initial assessment at LC site

Figure A4 - Bar chart comparing the main symptoms, displayed across the different PCFS scales

Figure A5 - GroC by PCFS and Groc By year of assessment GROC and vocational status

Figure A6 – Distribution of perceived trajectory of recovery using a GroC scale, according to year of initial assessment
